# Comparative Efficacy of Chinese Herbal Injections for Septic Shock: A Bayesian Network Meta-Analysis of Randomized Controlled Trials

**DOI:** 10.3389/fphar.2022.850221

**Published:** 2022-04-07

**Authors:** Peiying Huang, Yan Chen, Haobo Zhang, Bojun Chen, Shuai Zhao, Yuchao Feng, Sisi Lei, Qihua Wu

**Affiliations:** ^1^ The Second Clinical Medical College of Guangzhou University of Chinese Medicine, Guangzhou, China; ^2^ Guangdong Provincial Key Laboratory of Research on Emergency in Traditional Chinese Medicine, Clinical Research Team of Prevention and Treatment of Cardiac Emergencies with Traditional Chinese Medicine, Guangzhou, China; ^3^ Emergency Department of Guangdong Provincial Hospital of Traditional Chinese Medicine, Guangzhou, China

**Keywords:** Chinese herbal injections, Western medicine, septic shock, efficacy, Bayesian network meta-analysis

## Abstract

**Background:** Septic shock is associated with high morbidity and mortality. Studies have reported that Chinese herbal injections (CHIs) in combination with Western medicine (WM) were more favorable. However, the debate on optimal CHIs is ongoing. The objective of this study is to explore the comparative effectiveness of CHIs for septic shock.

**Methods:** We retrieved data from the English and Chinese databases with retrieval time from database inception to 30 September 2021. Network meta-analysis was performed, with evaluation of methodological quality among the included studies and assessment of strength of evidence among the outcomes.

**Results:** A total of 77 RCTs with 5,647 patients were included. All the studies were rated as some concerns. In terms of 28-days-mortality, Yiqifumai injection (YQFM)+WM, Shuxuetong injection (SXT)+WM, Xuebijing injection (XBJ)+WM, and Shenfu injection (SF)+WM were better than WM; YQFM + WM and SXT + WM were superior for Shenmai injection (SM)+WM; YQFM + WM was superior for SF + WM; YQFM + WM ranked first. Regarding ICU length of stay, SF + WM and XBJ + WM were better than WM; XBJ + WM was superior for SF + WM; XBJ + WM ranked first. Concerning hospital length of stay, Shenqifuzheng injection (SQFZ)+WM, Shengmai injection (SGM)+WM, and XBJ + WM had greater potential than WM and SF + WM; SQFZ + WM ranked first. As for SOFA score at 7-days, XBJ + WM and SF + WM were superior for WM; XBJ + WM was superior for SF + WM; XBJ + WM ranked first. Regarding procalcitonin level at 7-days, SF + WM, SM + WM, and Xiyanping injection (XYP)+WM were better than WM; XYP + WM was superior for SF + WM, SGM + WM, SM + WM, Danshen injection (DS)+WM, and XBJ + WM; XYP + WM ranked first. Concerning serum lactate level at 7-days, SF + WM and SM + WM were more effective than XBJ + WM and WM; SM + WM ranked first. The comparisons were rated as moderate (15.05%), low (40.86%), and very low quality (44.09%); the strength of evidence of ranking probability for hospital length of stay was low whereas the remaining outcomes were rated as very low.

**Conclusions:** CHIs combined with WM might have higher efficacies for septic shock than WM alone. YQFM, XBJ, SQFZ, XYP, SM, SGM, and SF may be the potential optimal CHIs for septic shock. More and better evidence is needed to validate the conclusions.

**Systematic Review Registration:** https://www.crd.york.ac.uk/prospero/display_record.php?, identifier CRD42021282958.

## Introduction

As a common critical disease in emergency department and intensive care unit (ICU), septic shock is a critical syndrome caused by a dysregulated host response to infection, accompanied by circulatory failure that is difficult to correct (Shankar-Hari et al., 2016; Singer et al., 2016). Although western prevention plans or treatment programs for septic shock were being continually updated (Levy et al., 2018; Evans et al., 2021), worldwide, morbidity and mortality associated with septic shock are still high. In 2012, a global estimate for septic shock incidence was 11 per 100,000 people annually (Jawad et al., 2012), which increased to 19.3 per 1,00,000 person-years in 2016 (Shankar-Hari et al., 2017). In addition, the mortality of septic shock ranged from 20 to 50% based on the evaluations (Levy et al., 2012; Quenot et al., 2013; Kaukonen et al., 2014). More seriously, with the outbreak of COVID-19, the morbidity and mortality of septic shock may increase further (Karakike et al., 2021).

Septic shock therapy has conventionally been dominated by anti-infective drugs, intravenous fluid, and vasopressors, all of which, however, still have shortcomings in reducing mortality of septic shock (Bauer et al., 2020). To make up for the deficiency of conventional treatment approaches, adjuvant therapies for septic shock have been constantly explored, such as hydrocortisone, ascorbic acid, as well as thiamine (Marik et al., 2017; Annane et al., 2018), whereas the therapeutic effects of them were still far from satisfactory. Indeed, there is another promising complementary treatment, namely traditional Chinese medicine, going back over 1,000 years and being accepted as one of the main types of therapy for septic shock in China (Unschuld, 1999; Wang et al., 2020). Chinese herbal injections (CHIs), as one of the most common dosage forms of traditional Chinese medicine, have been proved to exert therapeutic effects on patients with septic shock (Zhou et al., 2016; Li, 2018; Ha et al., 2019).

However, there is a wide variety of CHIs used for septic shock; how to choose the most appropriate one remains a problem for clinicians when facing septic shock patients, especially patients in different disease states. There are no studies thus far comparing different categories of CHIs used in the treatment of septic shock. Consequently, we searched all the randomized controlled trials (RCTs) of CHIs used to treat septic shock and initiated this network meta-analysis (NMA) to compare the efficacy among them, hoping to provide some advice for clinical practice.

## Methods

We have registered on the International Prospective Register of Systematic Reviews with registration number CRD42021282958. The study followed the PRISMA Extension Statement (Hutton et al., 2015). The full and detailed PRISMA checklist is provided in Supplementary File S1.

### Search Strategy

We searched both English-language electronic databases (PubMed, embase, Web of Science, and Cochrane Library) and Chinese-language electronic databases (China National Knowledge Infrastructure, Wanfang database, Weipu Journal database, and Chinese Biomedical Literature database). RCTs published from database inception through 30 September 2021, were searched. The search strategies are detailed in Supplementary File S2. All the search results were downloaded and imported into EndNote X9 software.

### Types of Studies

RCTs were the original studies we consented to the inclusion, in which randomized crossover trials were excluded if the effect size of the early phase was not available. We had no restrictions on language, country, date of publication, and stage of the RCTs.

### Types of Participants

Patients aged 18 years or older, with the diagnosis of septic shock, were considered. The diagnostic criteria for septic shock were as follows:

(1) Sepsis 1.0: Sepsis-related hypotension persists despite adequate fluid resuscitation (systolic blood pressure <90 mmHg or decreases ≥40 mmHg from baseline in the absence of other causes for hypotension). Patients who appear to be normotensive after vasopressor therapy should also be considered (Bone et al., 1992).
(2) Sepsis 2.0: Sepsis-related hypotension persists despite adequate fluid resuscitation (systolic blood pressure <90 mmHg, mean arterial pressure <60 mmHg, or decreases ≥40 mmHg from baseline in the absence of other causes of hypotension) (Levy et al., 2003).
(3) Sepsis 3.0: Despite adequate fluid resuscitation, patients have serum lactate level >2 mmol/L (>18 mg/dl) regardless of the absence of hypovolemia and require vasopressor therapy to maintain mean arterial blood pressure ≥65 mmHg (Singer et al., 2016).

In addition, there were no restrictions on the gender, nationality, ethnicity, or race of the patients. However, studies targeting patients with concurrent septic shock and severe profiles of comorbidities (i.e., cardiac arrest and advanced cancer) that most likely impact prognosis were excluded.

### Types of Interventions

We required the RCTs to have at least two interventions, i.e., CHIs plus Western medicine (WM) versus WM, or CHIs plus WM versus another type of CHIs plus WM. We requested that the CHIs should be used intravenously, but not restricted time of intervention, the configuration of the drug, frequency of medication, and treatment cycle.

### Types of Outcomes

The primary outcome of this study was 28-days-mortality. Secondary outcomes included the following:

(1) ICU length of stay.
(2) Hospital length of stay.
(3) Sequential Organ Failure Assessment (SOFA)score at day 7 after interventions.
(4) Procalcitonin level at day 7 after interventions.
(5) Serum lactate level at day 7 after interventions.
(6) Adverse drug reactions (ADRs).

### Data Extraction and Quality Assessment

According to the inclusion and exclusion criteria, two reviewers independently screened the original studies by reading titles, abstracts, and full texts. The titles, years of publication, first-authors, sample sizes, age and gender of participants, diagnostic criteria, interventions, trial duration, outcomes, ADRs, blinding, and random methods of the selected studies were extracted with a predesigned form and were further entered into Excel 356 software.

Another two reviewers implemented quality assessment independently. We used Version 2 of the Cochrane risk-of-bias tool for randomized trials (RoB 2) (Sterne et al., 2019) to evaluate the quality of the included RCTs through the following aspects: randomization process, deviations from intended interventions, missing outcome data, measurement of the outcome, and selection of the reported result. Item “overall bias” summarized the overall assessment of the above five items. Each item contains “low risk”, “high risk”, and “some concerns”, and an RCT was rated as “low risk” overall only if all items of it were assessed as “low risk”. The current version (22 August 2019) of RoB 2 was used to produce a risk of bias graph.

When any disagreements occurred in the process of data extraction or quality assessment, the operators negotiated first. If the discrepancies were still unresolved, a third reviewer intervened in the arbitration.

### Data Analysis

In this study, the program was analyzed by the Bayesian algorithm (Salanti, 2012). Pooled dichotomous-effect measures were expressed as pooled risk ratio (RR)with 95% confidence interval (CI) while pooled continuous variables were expressed as mean differences (MD) with 95%CI. The Markov Chain Monte Carlo methodology was adopted to construct the environment of analysis based on a random-effects model (Salanti et al., 2008; Mavridis and Salanti, 2013). Based on four Monte Carlo Markov Chains, we set the number of iterations as 200,000 and the first 10,000 were used for the annealing algorithm to eliminate the influence of the initial value. Brooks-Gelman-Rubin plots were used to evaluate the goodness-of-fit of the result (The median line and the 97.5% line tended to 1 after iteration, indicating that the model was stable) (Brooks and Gelman, 1998). If the goodness-of-fit of the result was still unsatisfactory, the number of iterations was further increased until the goodness-of-fit was satisfactory. Additionally, no closed loop was formed among the interventions, therefore, inconsistency in the NMA was unnecessary to be detected.

We produced league tables to express the comparisons between each pair of interventions and ranked the CHIs in each outcome by the ranking probabilities produced by surface under the cumulative ranking area curves (SUCRA)to find the most suitable CHIs (Dias et al., 2013). Moreover, the ranking probability of interventions shared by the primary outcome and each of the five secondary outcomes were aggregated into a comprehensive ranking respectively through cluster analyses.

Per-comparison *I* ^2^ was used to measure the heterogeneity between each pair of interventions while global *I* ^2^ was used to measure the overall heterogeneity. A higher value of the *I* ^2^ denotes a greater degree of heterogeneity (Higgins and Thompson, 2002). Furthermore, sensitivity analysis and subgroup analysis were applied to identify the sources of the heterogeneity and evaluate the robustness of the pooled effects. In addition, funnel plots were used to explore publication bias in the outcomes with greater than or equal to 10 RCTs (Stuck et al., 1998).

All analyses were conducted using R 4.1.1 (gemtc package: NMA, assessment of heterogeneity, ranking probability of SUCRA, subgroup analysis; meta package: sensitivity analysis of pairwise interventions) and STATA 14.0 (publication bias and cluster analysis).

### Grading the Quality of Evidence

We employed the Grades of Recommendation, Assessment, Development, and Evaluation (GRADE) to summarize the quality of evidence via two aspects, i.e., pairwise comparison and rank probability among the interventions in each outcome (Salanti et al., 2014). The GRADE has four grades: high, moderate, low, and very low. In this study, each item initially was rated as “high quality” and further downgraded through the following: study limitation, indirectness, heterogeneity/inconsistency, imprecision, and publication bias.

## Results

### Search Results

**FIGURE 1:**
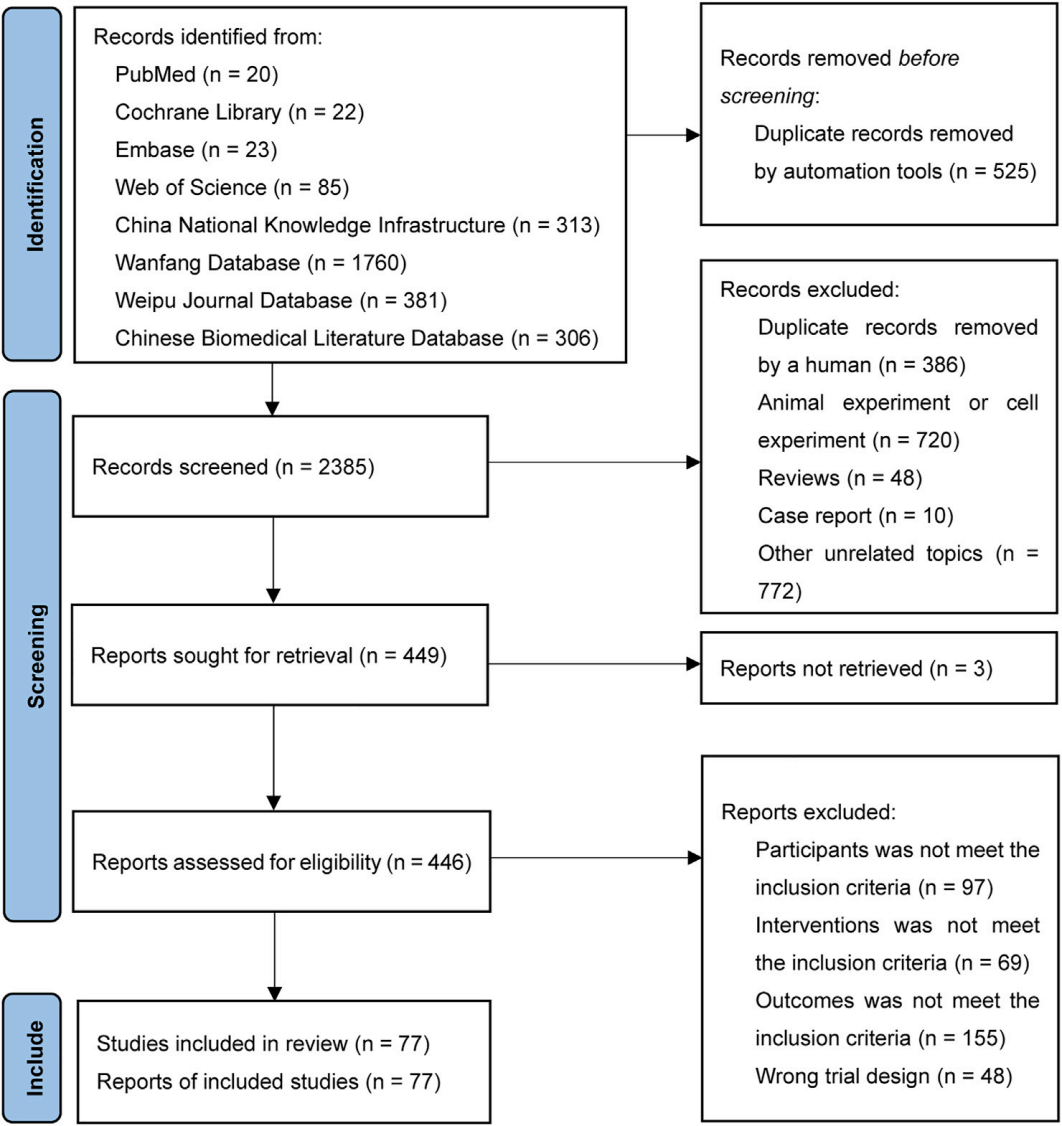
Flow chart for literature screening.

A total of 2,910 records were retrieved. After removing 911 duplicates, 1,550 records were removed in the first screening round by reading the titles and abstracts and 372 records were removed in the second screening round by reading the full text. Finally, 77 RCTs were included in our analysis with citations that are showed in Supplementary File S3. The flow chart of the literature search is provided in Figure 1.

### Included Studies and Characteristics

**FIGURE 2:**
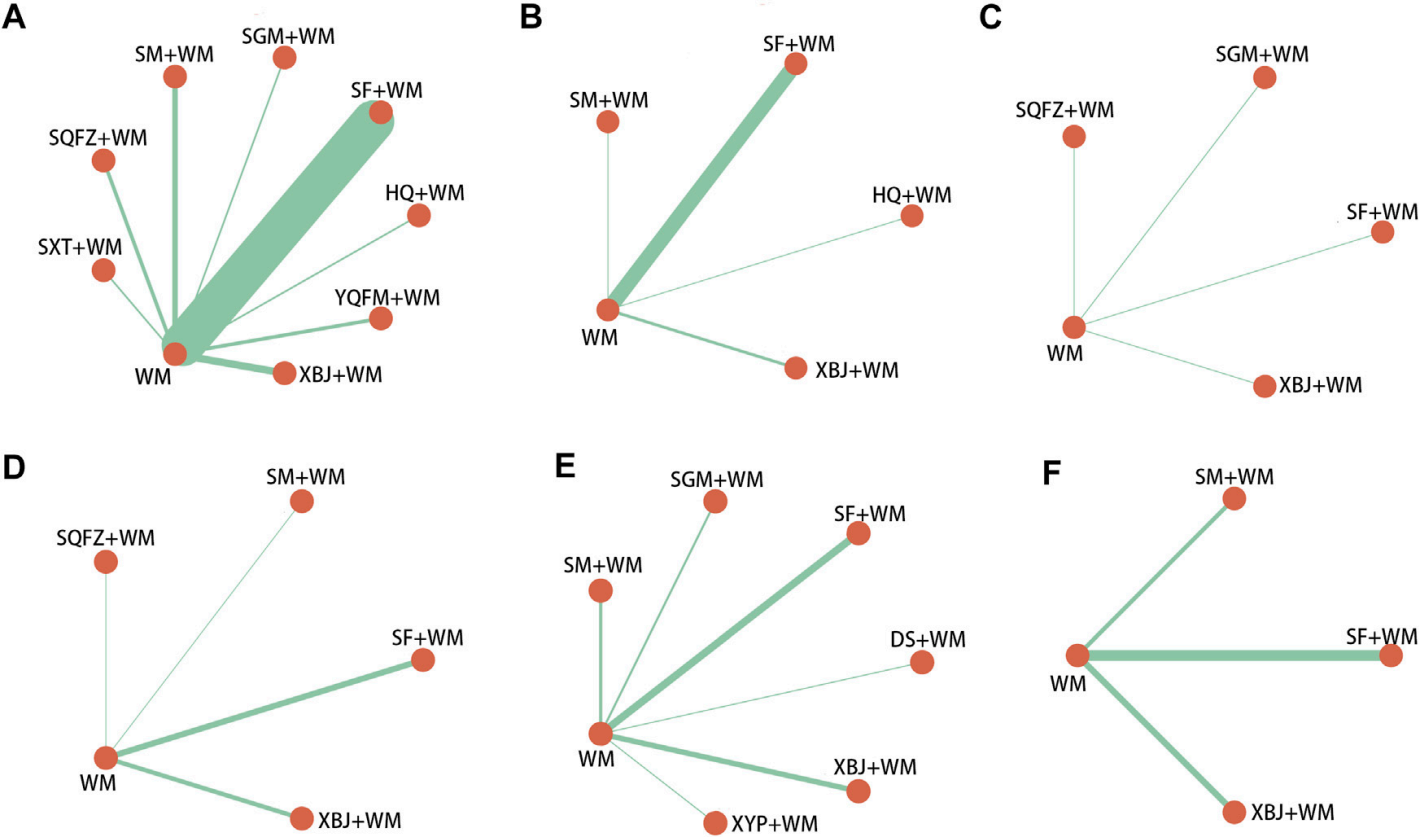
Network graph of different interventions **(A)** 28-days-motality **(B)** ICU length of stay **(C)** Hospital length of stay **(D)** SOFA score at day 7 after interventions **(E)** Procalcitonin level at day 7 after interventions **(F)** Serum lactate level at day 7 after interventions; WM, Western Medicine; SF, Shenfu injection; SM, Shenmai injection; SGM, Shengmai injection; XBJ, Xuebijing injection; YQFM, Yiqifumai injection; DS, Danshen injection; HQ, Huangqi injection; SXT, Shuxuetong injection; SQFZ, Shenqifuzheng injection; XYP, Xiyanping injection. The nodes were joined by different thickness lines which were generated to show whether there existed a direct relationship between treatments and the thickness was weighted according to the available direct evidence between them.

The selected studies consisted of 2 English-language studies and 75 Chinese-language studies, including 5,647 patients and 3,209 male patients among them (58.92%, sex ratios of three studies were unavailable). All the RCTs were two-arm studies with the time of publication from 2009 to 2021, involving 10 kinds of CHIs: Shenfu injection (SF, 35 RCTs), Shenmai injection (SM, eight RCTs), Shengmai injection (SGM, five RCTs), Xuebijing injection (XBJ, 20 RCTs), Yiqifumai injection (YQFM, 2 RCTs), Danshen injection (DS, one RCT), Huangqi injection (HQ, one RCT), Shuxuetong (SXT, one RCT), Shenqifuzheng (SQFZ, three RCTs), and Xiyanping injection (XYP, one RCT). Thirty-seven (48.05%), 20 (25.97%), 10 (12.99%), 16 (20.78%), 19 (24.68%), and 20 (25.97%) studies, individually, contributed to six outcomes: 28-days-mortality, ICU length of stay, hospital length of stay, SOFA score at 7-days, procalcitonin level at 7-days and serum lactate level at 7-days. The details of the included CHIs are summarized in Supplementary File S4, and the details of the selected studies are shown in Table 1. The network graphs of the interventions with different outcomes are depicted in Figure 2.

**TABLE 1:** Characteristics of the included studies.

Study ID	Sample size (E/C)	Sex (M/F)	Age (year, E/C)	Consistent Baseline	Definitions of Septic Shock	Intervention in CHIs Group*		Intervention in WM Group	Course of treatment (Days)	Outcomes	Adverse drug reactions
Li ML 2019	25/25	30/20	67.64 ± 14.49/68.84 ± 15.80	Y	Sepsis 2.0	SF 60 ml + 5%GS or 0.9%NS, ivgtt, qd		WM	7	①④	NR
Li MQ 2015	21/24	28/17	54.9 ± 14.7/57.5 ± 16.1	Y	Sepsis 2.0	SF 100 ml, 20 ml/h, ivvp, bid		WM	-	①②④	NR
Li Y 2016	102/97	124/75	54.0 ± 16.9/54.0 ± 16.9	Y	Sepsis 2.0	SF 30 ml/h for 3 h, ivvp for the first day, then 100 ml + 200 ml 0.9%NS, ivgtt, qd		WM	5	①②③	N
Xie RF 2016	25/25	30/20	57.3 ± 9.2/57.7 ± 8.9	Y	Sepsis 2.0	YQFM 5.2 g + 0.9%NS 500 ml, ivgtt, qd		WM	14	①	NR
Sun RQ 2020	40/40	55/25	59.38 ± 12.12/57.95 ± 13.64	Y	Sepsis 3.0	XBJ 100 ml + 0.9%NS 100 ml, ivgtt, bid		WM	7	①②③⑤	NR
Cao SX 2021	49/49	57/41	43.27 ± 6.17/45.36 ± 5.78	Y	Sepsis 2.0	XBJ 50 ml + 0.9%NS 100 ml, ivgtt, bid or tid		WM	7	⑤	NR
Chen DX 2020	35/35	48/22	60.1 ± 4.8/59.3 ± 4.6	Y	NR	XBJ 50 ml + 0.9%NS 100 ml, ivgtt, tid		WM	7	③	NR
Chen RJ 2017	30/30	32/28	50.20 ± 12.30/54.50 ± 14.30	Y	Sepsis 2.0	SF 60 ml + 0.9%NS 250 ml, ivgtt, qd		WM	7	④⑤	NR
Chen RJ 2015	20/20	24/16	54.6 ± 14.2/50.5 ± 10.5	Y	Sepsis 2.0	SF 60 ml + 0.9%NS 250 ml, ivgtt, qd		WM	7	⑤	NR
Chen S 2018	39/39	44/34	47.32 ± 5.29/47.24 ± 5.31	Y	Sepsis 2.0	XBJ 50 ml + 0.9%NS 100 ml, ivgtt, bid		WM	7	⑥	NR
Chen Z 2016	36/36	46/26	39.8 ± 2.7	Y	Sepsis 2.0	SF 100 ml + 5%GS 250 ml, ivgtt		WM	-	①②	NR
Cheng TC 2018	34/34	44/24	56.65 ± 8.17/57.33 ± 7.29	Y	Sepsis 2.0	SF 100 ml + 5%GS 250 ml, ivgtt, qd		WM	7	④⑤⑥	NR
Cui LC 2021	31/31	37/25	59.06 ± 4.37/59.89 ± 4.53	Y	Sepsis 2.0	SGM 60 ml + 5%GS 250 ml, ivgtt, qd		WM	7	⑤	Detailed description
Cui Y 2016	40/40	44/36	58.2 ± 12.0/59.1 ± 10.4	Y	Sepsis 2.0	SF 100 ml, 20 ml/h, ivvp, bid		WM	7	①②④	NR
Dong GY 2014	46/45	55/36	68.34/69.56	Y	Sepsis 1.0	SF 100 ml + 0.9%NS 250 ml, ivgtt, qd		WM	-	①	NR
Fan YX 2014	30/30	33/27	63.00 ± 4.37/62.86 ± 3.88	Y	NR	XBJ 50 ml + 0.9%NS 100 ml, ivgtt, bid		WM	7	③	NR
Gao DN 2017	35/32	32/35	64.85 ± 12.26/65.03 ± 13.95	Y	Sepsis 3.0	SQFZ 250 ml, ivgtt, qd		WM	10	④	NR
Heng JF 2013	32/35	41/26	45 ± 18.53	Y	Sepsis 2.0	XBJ 50 ml + 0.9%NS 100 ml, ivgtt, bid		WM	7	⑥	NR
Huang XX 2015	20/20	24/16	55 ± 6/57 ± 8	Y	Sepsis 2.0	SF 50 ml/h, 2 h, ivvp, qd		WM	7	①②⑥	NR
Lai ZZ 2018	25/25	30/20	55.4 ± 17.5/50.2 ± 13.6	Y	Sepsis 2.0	SF 100 ml, ivgtt, bid		WM	2	①②③	NR
Lei XY 2016	30/30	31/29	65.4 ± 13.1/64.5 ± 12.2	Y	Sepsis 2.0	SF 100 ml + 5%GS 250 ml, ivgtt, qd		WM	7	①⑥	NR
Li CL 2014	34/34	50/18	34.8 ± 19.2/6.5 ± 21.6	Y	Sepsis 2.0	SF 50 ml, ivgtt, qd		WM	-	③	NR
Li JY 2012	8/8	11/5	64.38 ± 6.05/70.25 ± 4.27	Y	Sepsis 2.0	SF 100 ml + 0.9%NS 200 ml, 150 m/h, ivvp, qd		WM	5	①②	N
Li JS 2020	13/10	13/10	67.38 ± 11.1/72.5 ± 9.68	Y	Sepsis 3.0	SF 100 ml + 5%GS 150 ml, ivgtt, qd		WM	5	①③	N
Li LW 2017	25/25	24/26	59.23 ± 11.34/59.23 ± 10.69	Y	Sepsis 2.0	XBJ 50 ml + 0.9%NS 100 ml, ivgtt, bid		WM	7	⑥	NR
Li Q 2018	18/20	18/20	53.67 ± 10.28/51.20 ± 10.08	Y	Sepsis 2.0	XBJ 50 ml + 0.9%NS 100 ml, ivgtt, tid		WM	-	⑥	NR
Lin B 2014	26/25	-	>18 years	Y	Sepsis 2.0	SGM 60 ml + 0.9%NS 250 ml, ivgtt, qd		WM	-	①	NR
Lin B 2019	101/97	125/73	51.13 + 8.38/51.46 ± 8.1	Y	Sepsis 2.0	XBJ 50 ml + 0.9%NS 100 ml, ivgtt, bid		WM	7	①④	NR
Liu H 2009	23/23	-	>18 years	Y	Sepsis 1.0	HQ 30 ml + 0.9%NS 100 ml, ivgtt, bid		WM	-	①②	NR
Liu ML 2017	42/40	43/39	60.47 ± 12.78/62.56 ± 10.79	Y	Sepsis 2.0	SF 100 ml + 0.9%NS 100 ml, ivgtt, qd		WM	7	①②④⑥	NR
Liu PF 2018	31/31	37/25	47.7 ± 6.3/47.6 ± 6.2	Y	Sepsis 3.0	SM 20–100 ml + 5%GS 250–500 ml, ivgtt, qd		WM	7	⑤	NR
Liu WR 2019a	51/51	64/38	75.2 ± 8.6/73.5 ± 8.1	Y	Sepsis 2.0	SM 100 ml, ivgtt, qd		WM	5	①②	N
Liu WR 2019b	36/36	46/26	52.87 ± 3.49/53.77 ± 3.63	Y	Sepsis 2.0	SM 100 ml, ivgtt, qd		WM	7	①	NR
Lu PJ 2014	26/26	30/22	54.48 ± 9.25/56.52 ± 8.68	Y	NR	SQFZ 250 ml, ivgtt, qd		WM	-	①③	NR
Lu D 2017	20/20	21/19	52.2 ± 16.4/49.2 ± 16.5	Y	Sepsis 2.0	SM 100 ml, ivgtt, qd		WM	7	⑤⑥	N
Luo RC 2009	26/26	32/20	23–76	Y	Sepsis 2.0	XBJ 100 ml, ivgtt, bid		WM	21	①	NR
Luo Y 2019	54/54	58/50	68.41 ± 3.17	Y	NR	SGM 60 ml + 5%GS 250 ml, ivgtt, qd		WM	7	⑤	NR
Ma JS 2013	30/30	37/23	50.7 ± 6.6/51.2 ± 6.3	Y	NR	SF 60 ml, 20 ml/h, ivvp, qd		WM	-	①②	NR
Meng QL 2018	40/40	43/37	69.6 ± 8.4/70.5 ± 9.3	Y	Sepsis 2.0	XYP, ivgtt		WM	-	⑤	NR
*Pan* Y 2020	35/35	44/26	51.63 ± 6.50/51.20 ± 6.14	Y	Sepsis 2.0	SF 60 ml + 5%GS 250 ml, ivgtt, qd		WM	7	⑤⑥	NR
Peng ZL 2021	29/29	28/30	56.85 ± 2.77/57.41 ± 3.25	Y	NR	SGM 70–90 ml + 10% GS 300 ml, ivgtt, qd		WM	14	③	Detailed description
Ren DH 2015	26/25	31/20	69.6 ± 13.6/68.9 ± 15.1	Y	Sepsis 2.0	SM 50 ml, ivgtt, qd		WM	14	①	NR
Sang ZZ 2019	50/54	-	18–65	Y	Sepsis 2.0	SF 100 ml, ivgtt, bid		WM	7	①②	NR
Shi BZ 2019	53/53	72/34	50.5 ± 13.2/50.3 ± 13.1	Y	Sepsis 2.0	SM 200 ml, ivgtt, qd		WM	5	④⑤⑥	NR
Shi YJ 2021	30/30	41/19	59.06 ± 7.28/58.93 ± 6.62	Y	Sepsis 2.0	SF 100 ml + 5%GS 100 ml, ivgtt, qd		WM	-	④	N
Tang ZL 2015	77/42	81/38	44.7 ± 32.6/43.9 ± 29.7	Y	Sepsis 2.0	SXT 6 ml + 0.9%NS 250 ml, ivgtt, qd		WM	7	①	NR
Wang DS 2011	17/17	19/15	42.0 ± 5.4/41.3 ± 5.2	Y	NR	DS 20 ml + 10%GS, ivgtt		WM	7	⑤	NR
Wang JY 2015	10/10	12/8	60.70 ± 10.30	Y	Sepsis 2.0	SF 50 ml + 5%GS 250 ml, ivgtt, qd		WM	7	⑥	NR
Wang L 2018	33/32	39/26	41.65 ± 7.26/41.49 ± 7.31	Y	Sepsis 2.0	XBJ 50 ml + 0.9%NS 100 ml, ivgtt, bid		WM	7	④⑥	NR
Wang ZC 2015	38/38	40/36	52.3	Y	NR	SF 100 ml + 0.9%NS/5%GS 250 ml, ivgtt, bid		WM	10	①	NR
Xiao YC 2017	36/35	45/26	65.72 ± 12.24/66.42 ± 13.75	Y	Sepsis 2.0	SF 100 ml + 5%GS 250 ml, ivgtt, qd		WM	5	①②	NR
Xie Q 2016	49/49	48/50	53.4 ± 12.3/52.8 ± 11.5	Y	Sepsis 2.0	XBJ 50 ml + 0.9%NS 100 ml ivgtt, bid		WM	14	②	NR
Xu R 2019	34/34	38/30	48.32 ± 8.76/49.12 ± 9.16	Y	Sepsis 3.0	SF 100 ml + 5%GS 250 ml, ivgtt, qd		WM	7	①	NR
Yan ZJ 2018	25/25	37/13	65.51 ± 1.62/65.44 ± 1.74	Y	NR	SF 100 ml + 5%GS 250 ml, ivgtt, qd		WM	7	⑤	NR
Yang YJ 2020	25/25	27/23	65.21 ± 2.57/65.78 ± 2.20	Y	NR	SF 100 ml, ivgtt, qd		WM	7	⑤	NR
Yao S 2015	20/20	25/15	63.3 ± 11.4/63.2 ± 6.6	Y	Sepsis 2.0	SF 100 ml + 10%GS 250 ml, ivgtt, qd		WM	15	①⑥	NR
Yin X 2018	35/31	46/20	64.1 ± 15.8/62.4 ± 19.8	Y	Sepsis 2.0	XBJ 50 ml ivgtt, bid		WM	5	①	NR
Zhang JJ 2014	30/30	36/24	56.5 ± 7.8	Y	Sepsis 2.0	XBJ 50 ml + 0.9%NS 100 ml ivgtt, bid		WM	7	②⑤	NR
Zhang JM 2019	58/58	69/47	55.28 ± 4.59/52.19 ± 5.52	Y	NR	SF 100 ml + 0.9%NS 100 ml, ivgtt, qd		WM	-	①②	NR
Zhang L 2016	72/72	95/49	65.87 ± 17.28/64.35 ± 18.19	Y	NR	SM 100 ml, ivgtt, qd		WM	7	⑥	NR
Zhang RM 2016	64/66	79/51	72.9 ± 7.6	Y	Sepsis 2.0	XBJ 60 ml, 20 ml/h, ivvp		WM	7	④	NR
Zhang SY 2017	36/35	39/32	71.43 ± 9.21/69.37 ± 10.35	Y	Sepsis 2.0	SF 100 ml + 5%GS 250 ml, ivgtt, qd		WM	7	①②④	NR
Zhang WM 2017	41/41	47/35	51.32 ± 4.57/50.89 ± 5.18	Y	NR	SM 60 ml + 0.9%NS 250 ml, ivgtt, qd		WM	7	⑥	NR
Zhang Y 2018	64/64	63/65	52.49 ± 3.52/53.12 ± 4.73	Y	Sepsis 2.0	YQFM 2.6–5.2 g + 5%GS 250 ml, ivgtt, qd		WM	-	①	N
Zhang YN 2017	58/58	71/45	41.87 ± 9.91/42.13 ± 9.86	Y	Sepsis 2.0	SF 100 ml + 5%GS 250 ml, ivgtt, qd		WM	7	⑤⑥	NR
Zhang YH 2016	30/30	29/31	62.73 ± 14.79/59.44 ± 12.25	Y	Sepsis 2.0	SQFZ 250 ml, ivgtt, qd		WM	5	①	NR
Zhao N 2020	98/98	94/102	52.65 ± 5.53/51.43 ± 4.94	Y	NR	SF 100 ml + 5%GS 250 ml, ivgtt, qd		WM	7	⑥	NR
Zhao WP 2019	37/37	48/26	54.91 ± 10.34/55.13 ± 10.77	Y	NR	XBJ 50 ml + 0.9%NS 100 ml, ivgtt, bid		WM	7	⑤⑥	NR
Zheng XS 2013	22/22	23/21	43.5 ± 4.9	Y	Sepsis 2.0	XBJ 50 ml + 0.9%NS 100 ml, ivgtt, bid		WM	7	⑤	NR
Zheng Y 2014	38/40	42/36	70.25 ± 9.56/69.48 ± 10.13	Y	Sepsis 2.0	SF 100 ml + 5%GS 250 ml, ivgtt, qd		WM	7	①⑥	N
Zhong J 2017	15/15	11/19	49.75 ± 5.83/49.04 ± 5.97	Y	NR	XBJ 50 ml + 0.9%NS 100ml, ivgtt, bid		WM	7	④	NR
Zhong JX 2019	38/38	41/35	58.98 ± 9.22/58.75 ± 9.14	Y	Sepsis 2.0	XBJ 50 ml + 0.9%NS 100 ml, ivgtt, qd		WM	7	④	NR
Zhong KL 2015	32/32	47/17	59.8 ± 14.1/59.4 ± 14.5	Y	Sepsis 2.0	SF 20 ml/h, ivvp, 200 ml/d		WM	-	②	NR
Zhou CL 2014	30/30	33/27	70.15 ± 3.45/69.43 ± 2.84	Y	Sepsis 2.0	SF 100 ml + 5%GS 250 ml, ivgtt, qd		WM	7	①	NR
Zhou L 2013	36/39	48/27	69.72 ± 13.4/67.35 ± 15.8	Y	Sepsis 2.0	SF 100 ml + 5%GS 250 ml, ivgtt, qd		WM	-	①②	NR
Zhou LQ 2016	44/36	43/37	50.81 ± 12.04/51.68 ± 13.47	Y	Sepsis 2.0	XBJ 100 ml + 0.9%NS 100 ml, ivgtt, bid		WM	7	④	NR
Zou H 2020	35/35	37/33	57.56 ± 2.77/57.13 ± 2.11	Y	NR	SGM 20–60 ml + 5%GS 250–500 ml, ivgtt		WM	7	③	NR

Note: E/C, experimental group/control group; M/F, male/female; CHIs, Chinese herbal injections; WM, western medicine; * CHIs, were in addition to the treatment of control group; NR, not reported; N, no; SF, shenfu injection; SM, shenmai injection; SGM, shengmai injection; XBJ, xuebijing injection; YQFM, yiqifumai injection; DS, danshen injection; HQ, huangqi injection; SXT, shuxuetong injection; SQFZ, shenqifuzheng injection; XYP, xiyanping injection; ①, 28-days mortality; ②, ICU, length of stay; ③, Hospital length of stay; ④, SOFA, score at day 7 after interventions; ⑤, Procalcitonin level at day 7 after interventions; ⑥, Serum lactate level at day 7 after interventions.

### Methodological Quality

**FIGURE 3:**
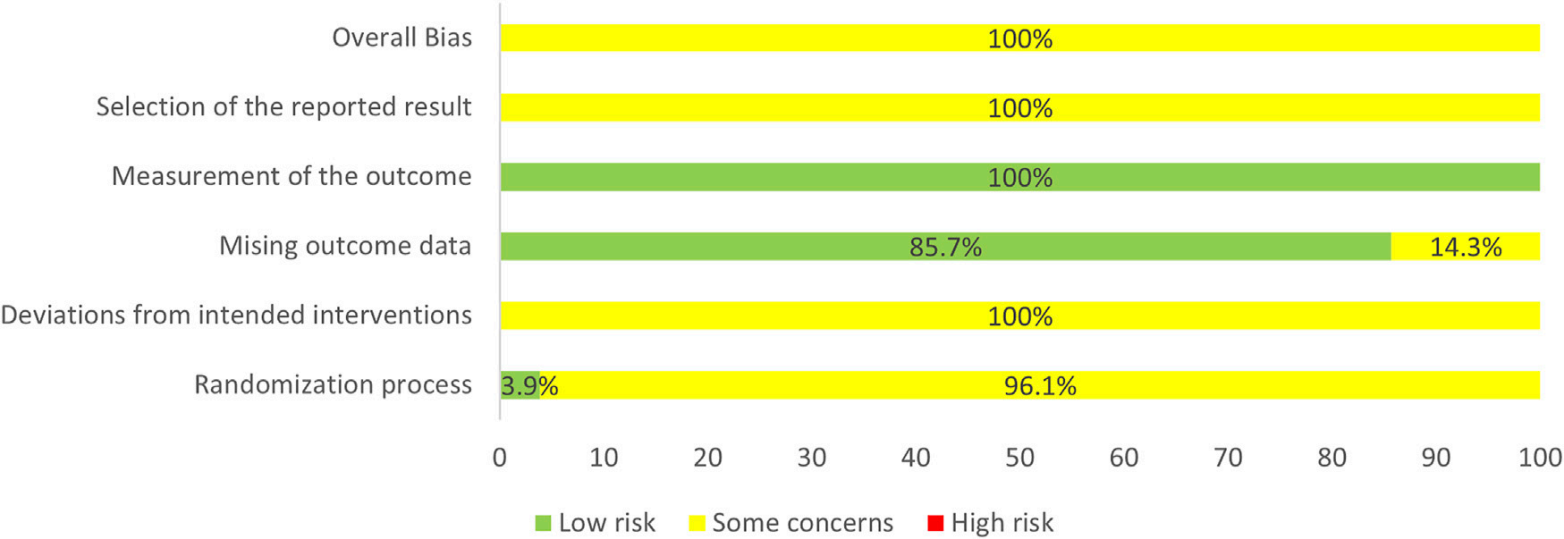
Assessment of risk bias.

In the selected RCTs, 35 RCTs (45.45%) did not mention specific random methods, 2 RCTs (2.6%) performed central randomization, one RCT (1.3%) performed block randomization, one RCT (1.3%) performed simple randomization via coin toss method, and 38 RCTs (49.35%) performing simple randomization via the table of random digits. Seventy-four RCTs (96.1%) did not state the details of allocation concealment, which were evaluated as “some concerns” in “randomization process”. One RCT reported no blinding was used while the remaining RCTs did not report blinding. We guessed that the remaining included studies were difficult to implement blinding and probably did not use appropriate analyses (i.e., intention-to-treat analyses or modified intention-to-treat analyses). Thus, the item, “deviations from intended interventions”, was rated as “some concerns”. In addition, 66 RCTs reported the number of patients who participated in the assessment of each outcome measure while 11 RCTs did not report, which resulted in 14.3% of the studies being rated as “some concerns” in the “missing outcome data”. “Measurement of the outcome” assessment was generally a “low risk of bias” as all the outcomes were obtained from objective measures. “Selection of the reported result” of all the RCTs were rated as “some concerns” because pre-specified protocols of the selected RCTs were unavailable, which made it impossible to assess whether the results were selectively reported. In general, the risk of bias of the selected RCTs was rated as “some concerns”. The results of the assessment of the risk of bias are presented in Figure 3.

### Network Meta-Analysis

In the Brooks-Gelman-Rubin plots, all the median lines and the 97.5% lines tended to 1, which indicated that all the model fits in the study were good. The Brooks-Gelman-Rubin plots are provided in Supplementary File S5.

### 28-Day Mortality

**FIGURE 4:**
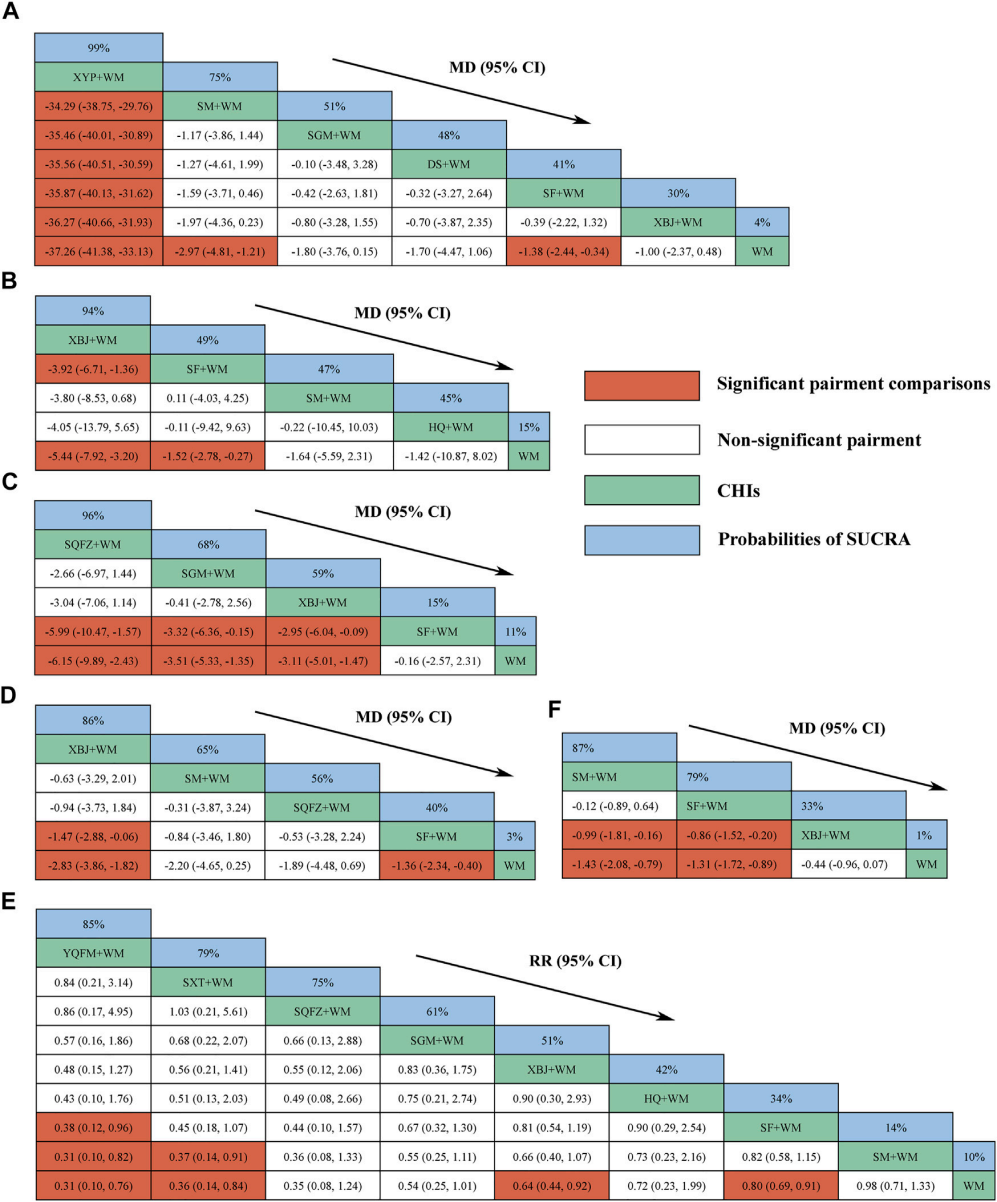
Relative effect sizes of efficacy at interventions in each outcome **(A)** Procalcitonin level at day 7 after interventions **(B)** ICU length of stay **(C)** Hospital length of stay **(D)** SOFA score at day 7 after interventions **(E)** 28-days-motality **(F)** Serum lactate level at day 7 after interventions; CHIs, Chinese herbal injections; WM, Western Medicine; SF, Shenfu injection; SM, Shenmai injection; SGM, Shengmai injection; XBJ, Xuebijing injection; YQFM, Yiqifumai injection; DS, Danshen injection; HQ, Huangqi injection; SXT, Shuxuetong injection; SQFZ, Shenqifuzheng injection; XYP, Xiyanping injection; SUCRA, surface under the cumulative ranking area curves. Highest probability of being the most efficient CHIs (With high SUCRA values) and Lowest probability of being the most efficient CHIs (With low SUCRA values).

Eight CHIs (YQFM, SXT, SQFZ, SGM, XBJ, HQ, SF, and SM) were involved in assessing 28-days mortality. According to the RR and 95%CI between all the pairwise interventions, YQFM + WM, SXT + WM, XBJ + WM, and SF + WM were superior for WM; YQFM + WM and SXT + WM were superior for SM + WM; YQFM + WM was superior for SF + WM; no such evident effect was observed with other pairwise interventions. Moreover, YQFM + WM, with the highest-ranking probability of SUCRA (85%), had the best effectiveness in reducing 28-days mortality, followed by SXT + WM (79%) and SQFZ + WM (75%). More details about the between-intervention differences and the rank probability of SUCRA are shown in Figure 4E.

### ICU Length of Stay

Four CHIs (XBJ, SF, SM, and HQ) were involved in reporting ICU length of stay. According to the MD and 95%CI between all the pairwise interventions, SF + WM and XBJ + WM were superior for WM; XBJ + WM was superior for SF + WM; no such evident effect was observed with other pairwise interventions. Moreover, XBJ + WM, with the highest-ranking probability of SUCRA (94%), had the best effectiveness in reducing ICU length of stay, followed by SF + WM (49%) and SM + WM (47%). More details about the between-intervention differences and the rank probability of SUCRA are shown in Figure 4B.

### Hospital Length of Stay

Four CHIs (SQFZ, SGM, XBJ, and SF) were involved in reporting hospital length of stay. According to the MD and 95%CI between all the pairwise interventions, SQFZ + WM, SGM + WM, and XBJ + WM were superior for WM and SF + WM; no such evident effect was observed with other pairwise interventions. Moreover, SQFZ + WM, with the highest-ranking probability of SUCRA (96%), had the best effectiveness in reducing hospital length of stay, followed by SGM + WM (68%) and XBJ + WM (59%). More details about the between-intervention differences and the rank probability of SUCRA are shown in Figure 4C.

### SOFA Score at Day 7 After Interventions

Four CHIs (XBJ, SM, SQFZ, and SF) were involved in reporting SOFA score at 7-days. According to the MD and 95%CI between all the pairwise interventions, XBJ + WM and SF + WM were superior for WM; XBJ + WM was superior for SF + WM; no such evident effect was observed with other pairwise interventions. Moreover, XBJ + WM, with the highest-ranking probability of SUCRA (86%), had the best effectiveness in reducing the SOFA score at 7-days, followed by SM + WM (65%) and SQFZ + WM (56%). More details about the between-intervention differences and the rank probability of SUCRA are shown in Figure 4D.

### Procalcitonin Level at Day 7 After Interventions

Six CHIs (XYP, SM, SGM, DS, SF, and XBJ) were involved in reporting procalcitonin level at 7-days. According to the MD and 95%CI between all the pairwise interventions, SF + WM, SM + WM, and XYP + WM were superior for WM; XYP + WM was superior for SF + WM, SGM + WM, SM + WM, DS + WM, and XBJ + WM; no such evident effect was observed with other pairwise interventions. Moreover, XYP + WM, with the highest-ranking probability of SUCRA (99%), had the best effectiveness in reducing procalcitonin level at 7-days, followed by SM + WM (75%) and SGM + WM (51%). More details about the between-intervention differences and the rank probability of SUCRA are shown in Figure 4A.

### Serum Lactate Level at Day 7 After Interventions

Three CHIs (SM, SF, and XBJ) were involved in reporting serum lactate level at 7-days. According to the MD and 95%CI between all the pairwise interventions, SF + WM and SM + WM were superior for XBJ + WM and WM; no such evident effect was observed with other pairwise interventions. Moreover, SM + WM, with the highest-ranking probability of SUCRA (87%), had the best effectiveness in reducing serum lactate level at 7-days, followed by SF + WM (79%) and XBJ + WM (33%). More details about the between-intervention differences and the rank probability of SUCRA are shown in Figure 4F.

### Adverse Drug Reactions

Ten RCTs (12.99%) reported ADRs, in which only SGM had ADRs (ADRs rate of 6.66%). The ADRs of SGM encompassed: 2 allergic dermatitides, three nausea and vomiting, one bloating, one palpitation, and one headache. No patient withdrew from the studies because of the ADRs.

### Cluster Analysis

**FIGURE 5:**
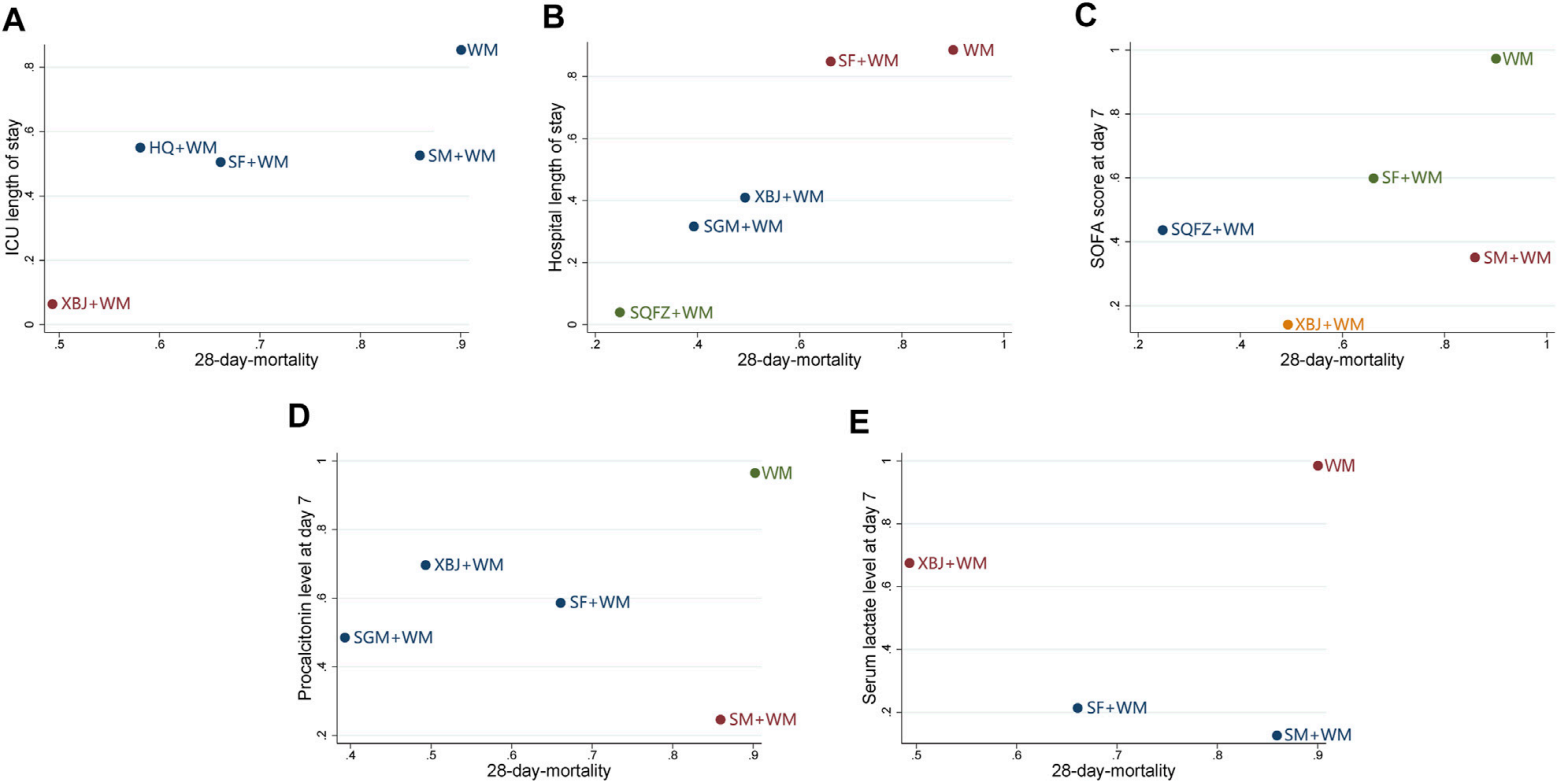
Cluster analysis plots **(A)** 28-days-motality (*x*-axis) and ICU length of stay (*y*-axis) **(B)** 28-days-motality (*x*-axis) and hospital length of stay (*y*-axis) **(C)** 28-days-motality (*x*-axis) and SOFA score at day 7 after interventions (*y*-axis) **(D)** 28-days-motality (*x*-axis) and procalcitonin level at day 7 after interventions (*y*-axis) **(E)** 28-days-motality (*x*-axis) and serum lactate level at day 7 after interventions (*y*-axis); WM, Western Medicine; SF, Shenfu injection; SM, Shenmai injection; SGM, Shengmai injection; XBJ, Xuebijing injection; HQ, Huangqi injection; SQFZ, Shenqifuzheng injection. Interventions with the same color belong to the same cluster, and interventions located in the lower-left corner indicate the optimal therapy for two different outcomes while located in the upper-right corner indicate the worst therapy.

Based on the SUCRA of the interventions shared by the pairwise outcomes, the cluster analysis was performed to integrate the effects of 28-days mortality with each of the first five secondary outcomes. As shown in Figure 5, XBJ + WM, SQFZ + WM, SGM + WM, SM + WM, and SF + WM achieved similarly superior effects over the others, and WM alone yielded the worst result.

### Publication Bias

**FIGURE 6:**
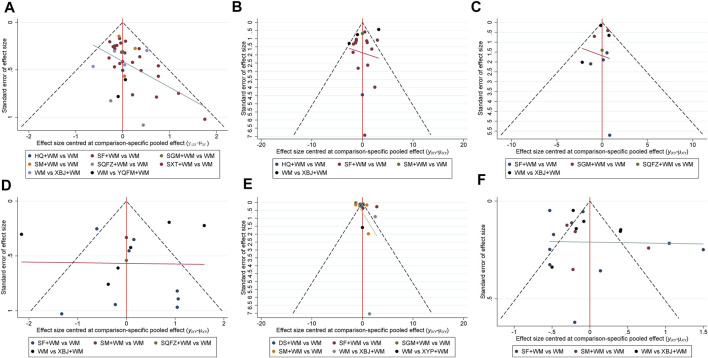
Funnel plots **(A)** 28-days-motality **(B)** ICU length of stay **(C)** Hospital length of stay **(D)** SOFA score at day 7 after interventions **(E)** Procalcitonin level at day 7 after interventions **(F)** Serum lactate level at day 7 after interventions; WM, Western Medicine; SF, Shenfu injection; SM, Shenmai injection; SGM, Shengmai injection; XBJ, Xuebijing injection; YQFM, Yiqifumai injection; DS, Danshen injection; HQ, Huangqi injection; SXT, Shuxuetong injection; SQFZ, Shenqifuzheng injection; XYP, Xiyanping injection.

Regarding the funnel charts of SOFA score at 7-days and serum lactate level at 7-days, the angle between the correction guideline and the centerline was large, which suggested the existence of potential publication bias. By contrast, the funnel charts showed unremarkable asymmetry on both sides of the centerline in 28-days mortality, ICU length of stay, hospital length of stay, and procalcitonin level at 7-days suggesting that it had no publication bias. The funnel charts are shown in Figure 6.

### Heterogeneity, Sensitivity Analysis, and Subgroup Analysis

As shown in Supplementary File S6, global *I* ^2^ was 0.0, 77.8, 14.1, 91.1, 99.6, and 93.5% for 28-days-mortality, ICU length of stay, hospital length of stay, SOFA score at 7-days, procalcitonin level at 7-days, and serum lactate level at 7-days, respectively. The five secondary outcomes had significant heterogeneity in the pairwise comparisons: SF + WM versus WM in “procalcitonin level at 7-days” (*I* ^2^ = 95.8%) and “serum lactate level at 7-days” (*I* ^2^ = 93.6%), SM + WM versus WM in “procalcitonin level at 7-days” (*I* ^2^ = 94.2%) and “serum lactate level at 7-days” (*I* ^2^ = 83.9%), SGM + WM versus WM in “hospital length of stay” (*I* ^2^ = 71.5%) and “procalcitonin level at 7-days” (*I* ^2^ = 74.3%), XBJ + WM versus WM in “ICU length of stay” (*I* ^2^ = 96.2%), “SOFA score at 7-days” (*I* ^2^ = 92.9%), “procalcitonin level at 7-days” (*I* ^2^ = 86.9%) and “serum lactate level at 7-days” (*I* ^2^ = 84.8%). Sensitivity analysis suggested that no selected literature was the source of the heterogeneity, and the pooled outcomes were steady. The results of sensitivity analysis are shown in Supplementary File S7. Additionally, subgroup analysis was performed in the selected studies which adopted septic shock in Sepsis 2.0 as the diagnostic criteria (the number of studies that used diagnostic criteria from Sepsis 1.0 and Sepsis 3.0 was too small to execute subgroup analysis). The subgroup analysis indicated some dissimilarities from the overall results: In terms of 28-days-mortality, XBJ + WM were significantly more effective than SF + WM and SM + WM; regarding ICU length of stay, no differences were found between XBJ + WM versus SF + WM/SF + WM versus WM; concerning SOFA score at day 7 after interventions, no discrepancies were observed between XBJ + WM versus SF + WM. The results are shown in Supplementary File S8.

### GRADE Evaluation of the Strength of Evidence

In the current study, the GRADE indicated the strength of evidence ranged from very low to moderate whereas most of the pairwise comparisons were rated as low (38, 40.86%) and very low (41, 44.09%), with only 14 (15.05%) comparisons being rated as moderate. In terms of ranking probability, the quality of evidence was low for hospital length of stay while the remaining outcomes were all rated as very low. The results of GRADE are detailed in Supplementary File S9.

## Discussion

Different from a conventional pairwise meta-analysis which can only compare two treatment formats at a time, NMA, a mixed treatment comparison or multiple treatments comparison meta-analysis, can compare the effects of greater than or equal to two treatments and allow ranking of different treatments by combining direct and indirect evidence (Caldwell et al., 2005; Li et al., 2011; Mills et al., 2012). In the current study, a Bayesian framework was used to conduct the model fitting of NMA, comparing ten CHIs in the treatment of septic shock. As showed in the results, YQFM, XBJ, SQFZ, SM, XYP, and SGM combined with WM demonstrated better outcomes compared with other CHIs combined with WM or WM alone.

In traditional Chinese medicine, septic shock belongs to “collapse syndrome”, with clinical symptoms such as apathy or even coma, pale, cold extremities, respiratory weakness, sweat profusely, and weak pulse (Liang et al., 2021). Physicians of traditional Chinese medicine consider that the disease is caused by the pathogenic Qi assaulting the human body and the vital Qi of the human body losing rapidly (Wang, 2015). Therapeutic approaches principally encompassing restoring the Yang, supporting the Healthy Energy, and expulsing the pathogenic Qi are used to alleviate the condition (Liang et al., 2021). Based on the theoretical context, traditional formulations such as Shengmaisan Decoction, Sini Decoction, and Xuefuzhuyu Decoction are utilized in clinical practice (Liang et al., 2021), involving *Panax ginseng* C. A. Mey [Araliaceae; Ginseng Radix et Rhizoma Rubra], *Aconitum carmichaeli* Debeaux [Ranunculaceae; Aconiti Lateralis Radix Praeparata], *Ophiopogon japonicus* (Thunb.) Ker-Gawl [Asparagaceae; Radix Ophiopogonis], *Schisandra chinensis* (Turcz.) Baill [Schisandraceae; Schisandrae Chinensis Fructus], *Salvia miltiorrhiza* Bunge [Lamiaceae; Salviae Miltiorrhizae Radix et Rhizoma], *Astragalus mongholicus* Bunge [Fabaceae; Astragali Radix], and so on. However, the oral mode of administration was the main modality of drug administration in traditional Chinese medicine previously, which might associate with inadequate bioavailability and slow occurrence. CHIs, the injections made of active ingredients in Chinese medicine compounds or single Chinese medicine and are used intravenously, nonetheless, might have a faster onset of action and better utilization (Zhang, 2016). This means of drug administration is probably more suitable for septic shock treatment.

In our study, the efficacy of septic shock treatment could be further increased by combined use with CHIs, which was similar to other pairwise meta-analyses (Zhou et al., 2016; Li, 2018; Ha et al., 2019). Although the full mechanism of action of CHIs for septic shock remained unclear, partial potential mechanisms of action were elucidated presently. Animal experiments demonstrated that for lipopolysaccharide-induced shock rats, YQFM could decrease cerebral venule albumin leakage and cerebrovascular hyperpermeability (Li D. T. et al., 2019), reduce the content of inflammatory factors in the lungs which result in lung injury (Xia et al., 2018), and inhibit the exudation of mesenteric venules as well as their local inflammation (Yuan et al., 2011; Ayididaer et al., 2021), all of which, might be attributed to the ginsenoside Rb1 and Sch isandrin incorporating in YQFM. In addition, the main pharmacological actions of XBJ include inhibiting the expression level of TNF-α, IL-1, IL-6, IL-8, IL-17, NF-κB, ET-1, tissue factor, macrophage migration inhibitory factor, malondialdehyde, and myeloperoxidase, reducing the apoptosis rate of immune cells, promoting the expression level of IL-10, endothelial nitric oxide synthase, superoxide dismutase, and glutathione peroxidase, enhancing Treg apoptosis, polarizing the immune response from Th2 to Th1, downregulating the expression of the TLR4/NF-κB signaling pathway, restoring the balance of the matrix metalloproteinase/tissue inhibitors of metalloproteinase ratio (Li et al., 2021). Regarding SQFZ, animal experiments confirmed that the drug could alleviate the acute lung injury induced by lipopolysaccharide in shock rats, of which the mechanism might relate to reducing the level of TNF-α and down-regulating the expression of chemokines fractalkine mRNA in lung tissue or inducing the expression of heat shock protein-70 (Wang et al., 2007; Liang et al., 2016). As for SM, the drug protecting the cardiomyocytes and kidney cells of septic rats by up-regulating Bcl-2 protein and down-regulating Bax protein has been well established (Lin et al., 2013a; Lin et al., 2013b), and it has been confirmed that SM could inhibit the expression of inflammatory mediators (e.g., IL-6) and increase serum IgG level in an animal experiment (He et al., 2014). Additionally, as showed in this study, XYP exerted a meaningful reduction in procalcitonin level, which might inextricably link to its inhibiting ability of NF-κB, thus exerting functions of anti-inflammatory and immunomodulatory (Zhang and Cui, 2018). Besides, a mouse experiment observed a downward trend of INF-γ, TNF-α, and IL-2 in SGM treating mice, accompanied by increasing Occludin and decreasing MLCK protein compared with the model group (Lu et al., 2021), which indicated the relation between SGM, and septic shock might intimately relate to the proteins and inflammatory factors mentioned above.

In addition to the therapeutic effects of CHIs, ADRs should also be considered. From the descriptive results of our study, the ADRs only appeared in SGM and were non-fatal. Nevertheless, it was worth noting that the results might not be very persuasive as only ten studies in our study reported the events. Compared to other dosage forms of traditional Chinese medicine, CHIs have a higher ADRs rate; the drugs are more likely to have new or serious adverse effects than other types of injections (Li H. et al., 2019). Studies based on large sample sizes presented that the ADRs rate of YQFM, XBJ, SQFZ, SM, XYP and SGM were 0.176%–0.2%, 0.3%, 1.35%–1.53, 0.1, 2.1, and 0.8% separately (Zheng et al., 2006; Li et al., 2009; Hu, 2012; Ding et al., 2015; Chen and Zhu, 2018; Cao et al., 2019; Li K. et al., 2019; Zheng et al., 2019). The ADRs generally consisted of non-fatal events while anaphylactoid reaction or anaphylactic shock associated with a high risk of causing deaths (Li H. et al., 2019). A systematic review summarized previous studies and concluded the influencing factors of ADRs in CHIs: individual patient characteristics, characterizations of CHIs, pharmaceutical excipients, vehicles, and rational drug uses based on the theory of traditional Chinese medicine (Wang et al., 2018). Notably, risk factors for ADRs may be different in various CHIs. For example, drip rate exceeding 40 drops/min will increase the ADRs rate of YQFM (Cao et al., 2019); vehicle, dosage, patient age, drug combination, irrigating syringe, and fluid dripping were associated with ADRs of XBJ (Wang et al., 2019; Zheng et al., 2019); irrational compatibility and dosages may be potential risk factors for SM (Zhang et al., 2010). Anyhow, existing evidence showed that the ADRs of CHIs were mostly related to clinical irrational medicine use (Liu et al., 2018; Tan et al., 2019). It seems particularly important, therefore, to advocate the standard use of CHIs by clinicians (Xie et al., 2013).

## Limitation

Although we have evaluated CHIs from many aspects, there are still some limitations. First, due to the limited number of RCTs involving the studies of YQFM (2 RCTs), HQ (1 RCT), SXT (1 RCT), DS (1 RCT), and XYP (1 RCT), the pooled outcomes might not be sufficiently convincing. Second, the promotion of the results may be restricted because all the included RCTs were conducted in China. Third, fewer selected studies 5) used the diagnostic criteria for septic shock in Sepsis 3.0 and this might influence the applicability of the results. Finally, the quality of evidence for most outcomes was low and need further evidence. However, our findings still have implications for the management of septic shock.

## Conclusion

The results of our study showed that CHIs combined with WM were more effective than WM alone in the treatment of septic shock. YQFM, XBJ, SQFZ, XYP, SM, SGM, and SF deserve more attention when treating septic shock patients. The quality of evidence for most outcomes was low, therefore, high-quality RCTs are needed to confirm the conclusions.

## Data Availability

The original contributions presented in the study are included in the article/Supplementary Material, further inquiries can be directed to the corresponding authors.
