# Supplementary material for: Comparative Efficacy of Chinese Herbal Injections for Septic Shock: A Bayesian Network Meta-Analysis of Randomized Controlled Trials
Source: Front Pharmacol. 2022 Apr 7;13:850221. doi: 10.3389/fphar.2022.850221 (PMC9022100; doi:10.3389/fphar.2022.850221)
Supplement: Supplementary file 1 [file DataSheet1.docx]

Supplementary Material

# File 1: PRISMA checklist for network meta-analysis.

| **Section/topic** | **#** | **Checklist item** | **Reported on page #** |
| --- | --- | --- | --- |
| **TITLE** | | |  |
| Title | 1 | Identify the report as a systematic review incorporating a network meta-analysis (or related form of  meta-analysis). | 1 |
| **ABSTRACT** | | |  |
| Structured summary | 2 | Provide a structured summary including, as applicable:  Background: main objectives  Methods: data sources; study eligibility criteria, participants, and interventions; study appraisal; and synthesis methods, such as network meta-analysis.  Results: number of studies and participants identified; summary estimates with corresponding confidence/credible intervals; treatment rankings may also be discussed. Authors may choose to summarize pairwise comparisons against a chosen treatment included in their analyses for brevity.  Discussion/Conclusions: limitations; conclusions and implications of findings.  Other: primary source of funding; systematic review registration number with registry name. | 1-2 |
| **INTRODUCTION** | | |  |
| Rationale | 3 | Describe the rationale for the review in the context of what is already known, including mention of why a network meta-analysis has been conducted | 2 |
| Objectives | 4 | Provide an explicit statement of questions being addressed with reference to participants, interventions, comparisons, outcomes, and study design (PICOS). | 2 |
| **METHODS** | | |  |
| Protocol and registration | 5 | Indicate if a review protocol exists and if and where it can be accessed (e.g., Web address), and, if available, provide registration information including registration number. | 2 |
| Eligibility criteria | 6 | Specify study characteristics (e.g., PICOS, length of follow-up) and report characteristics (e.g., years considered, language, publication status) used as criteria for eligibility, giving rationale. Clearly describe eligible treatments included in the treatment network and note whether any have been clustered or merged into the same node (with justification). | 2-3 |
| Information sources | 7 | Describe all information sources (e.g., databases with dates of coverage, contact with study authors to identify additional studies) in the search and date last searched. | 2 |
| Search | 8 | Present full electronic search strategy for at least one database, including any limits used, such that it could be repeated. | 2 |
| Study selection | 9 | State the process for selecting studies (i.e., screening, eligibility, included in systematic review, and, if applicable, included in the meta-analysis). | 2 |
| Data collection process | 10 | Describe method of data extraction from reports (e.g., piloted forms, independently, in duplicate) and any processes for obtaining and confirming data from investigators. | 3 |
| Data items | 11 | List and define all variables for which data were sought (e.g., PICOS, funding sources) and any assumptions and simplifications made. | 3 |
| Geometry of the network | S1 | Describe methods used to explore the geometry of the treatment network under study and potential biases related to it. This should include how the evidence base has been graphically summarized for presentation, and what characteristics were compiled and used to describe the evidence base to readers | 3 |
| Risk of bias within individual studies | 12 | Describe methods used for assessing risk of bias of individual studies (including specification of whether this was done at the study or outcome level), and how this information is to be used in any data synthesis. | 3 |
| Summary measures | 13 | State the principal summary measures (e.g., risk ratio, difference in means). Also describe the use of additional summary measures assessed, such as treatment rankings and surface under the cumulative ranking curve (SUCRA) values, as well as modified approaches used to present summary findings from meta-analyses. | 3 |
| Planned methods of analysis | 14 | Describe the methods of handling data and combining results of studies for each network meta-analysis. This should include, but not be limited to: Handling of multigroup trials; Selection of variance structure; Selection of prior distributions in Bayesian analyses; and Assessment of model fit. | 3 |
| Assessment of inconsistency | S2 | Describe the statistical methods used to evaluate the agreement of direct and indirect evidence in the treatment network(s) studied. Describe efforts taken to address its presence when found. | 3 |
| Risk of bias across studies | 15 | Specify any assessment of risk of bias that may affect the cumulative evidence (e.g., publication bias, selective reporting within studies) | 3 |
| Additional analyses | 16 | Describe methods of additional analyses if done, indicating which were prespecified. This may include, but not be limited to, the following: Sensitivity or subgroup analyses; Meta-regression analyses; Alternative formulations of the treatment network; and Use of alternative prior distributions for Bayesian analyses (if applicable). | 3 |
| **RESULTS** | | |  |
| Study selection | 17 | Give numbers of studies screened, assessed for eligibility, and included in the review, with reasons for exclusions at each stage, ideally with a flow diagram. | 3-4 |
| Presentation of network structure | S3 | Provide a network graph of the included studies to enable visualization of the geometry of the treatment network. | 4, 7 |
| Summary of network geometry | S4 | Provide a brief overview of characteristics of the treatment network. This may include commentary on the abundance of trials and randomized patients for the different interventions and pairwise comparisons in the network, gaps of evidence in the treatment network, and potential biases reflected by the network structure. | 4 |
| Study characteristics | 18 | For each study, present characteristics for which data were extracted (e.g., study size, PICOS, follow-up period) and provide the citations. | 4-5 |
| Risk of bias within studies | 19 | Present data on risk of bias of each study and, if available, any outcome level assessment. | 4, 6 |
| Results of individual studies | 20 | For all outcomes considered (benefits or harms), present, for each study: 1) simple summary data for each intervention group, and 2) effect estimates and confidence intervals. Modified approaches may be needed to deal with information from larger networks. | 6 |
| Synthesis of results | 21 | Present results of each meta-analysis done, including confidence/credible intervals. In larger networks, authors may focus on comparisons versus a particular comparator (e.g., placebo or standard care), with full findings presented in an appendix. League tables and forest plots may be considered to summarize pairwise comparisons. If additional summary measures were explored (such as treatment rankings), these should also be presented. | 6 |
| Exploration for inconsistency | S5 | Describe results from investigations of inconsistency. This may include such information as measures of model fit to compare consistency and inconsistency models, P values from statistical tests, or summary of inconsistency estimates from different parts of the treatment network. | / |
| Risk of bias across studies | 22 | Present results of any assessment of risk of bias across studies for the evidence base being studied. | 10 |
| Results of additional analyses | 23 | Give results of additional analyses, if done (e.g., sensitivity or subgroup analyses, meta-regression  analyses, alternative network geometries studied, alternative choice of prior distributions for  Bayesian analyses, and so forth). | 7, 10 |
| **DISCUSSION** | | |  |
| Summary of evidence | 24 | Summarize the main findings, including the strength of evidence for each main outcome; consider their relevance to key groups (e.g., health care providers, researchers, and policymakers). | 10 |
| Limitations | 25 | Discuss limitations at study and outcome level (e.g., risk of bias), and at review level (e.g., incomplete retrieval of identified research, reporting bias). Comment on the validity of the assumptions, such as transitivity and consistency. Comment on any concerns regarding network geometry (e.g., avoidance of certain comparisons). | 11 |
| Conclusions | 26 | Provide a general interpretation of the results in the context of other evidence, and implications for future research. | 11 |
| **FUNDING** | | |  |
| Funding | 27 | Describe sources of funding for the systematic review and other support (e.g., supply of data); role of funders for the systematic review. This should also include information regarding whether funding has been received from manufacturers of treatments in the network and/or whether some of the authors are content experts with professional conflicts of interest that could affect use of treatments in the network. | 11 |

# File 2: Search strategy for network meta-analysis.

## Search strategy of China National Knowledge Infrastructure.

| No. | Search items |
| --- | --- |
| #1 | SU %= '感染性休克' OR SU %= '脓毒症休克' OR SU %= '脓毒性休克' OR SU %= '中毒性休克' OR SU %= '内毒素性休克' |
| #2 | SU %= '益气复脉' OR SU %= '益气扶正' OR SU %= '血必净' OR SU %= '参麦' OR SU %= '参附' OR SU %= '生脉' OR SU %= '醒脑静' OR SU %= '丹参' OR SU %= '参芪扶正' OR SU %= '疏血通' OR SU %= '黄芪' OR SU %= '痰热清' OR SU %= '热毒宁' OR SU %= '喜炎平' OR SU %= '清开灵' |
| #3 | FT = '随机' |
| #4 | #1 AND #2 AND #3 |

## Search strategy of Wanfang Database.

| No. | Search items |
| --- | --- |
| #1 | 主题:(感染性休克) or 主题:(脓毒症休克) or 主题:(脓毒性休克) or 主题:(中毒性休克) or 主题:(内毒素性休克) |
| #2 | 主题:(益气复脉) or 主题:(益气扶正) or 主题:(血必净) or 主题:(参麦) or 主题:(参附) or 主题:(生脉) or 主题:(醒脑静) or 主题:(丹参) or 主题:(参芪扶正) or 主题:(疏血通) or 主题:(黄芪) or 主题:(痰热清) or 主题:(热毒宁) or 主题:(喜炎平) or 主题:(清开灵) |
| #3 | 全部:(随机) |
| #4 | #1 AND #2 AND #3 |

## Search strategy of Chinese Biomedical Literature Database.

| No. | Search items |
| --- | --- |
| #1 | "感染性休克"[常用字段:智能] OR "脓毒症休克"[常用字段:智能] OR "脓毒性休克"[常用字段:智能] OR "中毒性休克"[常用字段:智能] OR "内毒素性休克"[常用字段:智能] |
| #2 | "醒脑静"[常用字段:智能] OR "丹参"[常用字段:智能] OR "参芪扶正"[常用字段:智能] OR "疏血通"[常用字段:智能] OR "黄芪"[常用字段:智能] OR "痰热清"[常用字段:智能] OR "热毒宁"[常用字段:智能] OR "喜炎平"[常用字段:智能] OR "清开灵"[常用字段:智能] OR"益气复脉"[常用字段:智能] OR "益气扶正"[常用字段:智能] OR "血必净"[常用字段:智能] OR "参麦"[常用字段:智能] OR "参附"[常用字段:智能] OR "生脉"[常用字段:智能] |
| #3 | "随机"[全部字段:智能]) |
| #4 | #1 AND #2 AND #3 |

## Search strategy of Weipu Journal Database.

| No. | Search items |
| --- | --- |
| #1 | M=(感染性休克 OR 脓毒症休克 OR 脓毒性休克 OR 中毒性休克 OR 内毒素性休克) |
| #2 | M=(益气复脉 OR 益气扶正 OR 血必净 OR 参麦 OR 参附 OR 生脉 OR 醒脑静 OR 丹参 OR 参芪扶正 OR 疏血通 OR 黄芪 OR 痰热清 OR 热毒宁 OR 喜炎平 OR 清开灵) |
| #3 | U=(随机) |
| #4 | #1 AND #2 AND #3 |

## Search strategy of Pubmed.

| No. | Search items |
| --- | --- |
| #1 | Shock, Septic [MeSH Terms] |
| #2 | Shock, Septic[Title/Abstract] OR Septic Shock[Title/Abstract] OR Shock, Toxic[Title/Abstract] OR Toxic Shock Syndrome[Title/Abstract] OR Shock Syndrome, Toxic[Title/Abstract] OR Toxic Shock Syndromes[Title/Abstract] OR Toxic Shock[Title/Abstract] OR Shock, Endotoxic[Title/Abstract] OR Endotoxin Shock[Title/Abstract] OR Endotoxin Shocks[Title/Abstract] OR Shock, Endotoxin[Title/Abstract] OR Shocks, Endotoxin[Title/Abstract] OR Sepsis Shock[Title/Abstract] OR Infection Shock[Title/Abstract] OR Infectious Shock[Title/Abstract] OR Infective Shock[Title/Abstract] |
| #3 | #1 OR #2 |
| #4 | Chinese Herbal Injection[Title/Abstract] OR Chinese Herbal Injections[Title/Abstract] OR xingnaojing[Title/Abstract] OR danshen[Title/Abstract] OR shenqifuzheng[Title/Abstract] OR shuxuetong[Title/Abstract] OR huangqi[Title/Abstract] OR tanreqing[Title/Abstract] OR reduning[Title/Abstract] OR xiyanping[Title/Abstract] OR qingkailing[Title/Abstract] OR yiqifuzheng[Title/Abstract] OR yiqifumai[Title/Abstract] OR xuebijing[Title/Abstract] OR shenfu[Title/Abstract] OR shenmai[Title/Abstract] OR shengmai[Title/Abstract] |
| #5 | Controlled Clinical Trial [Publication Type] OR Randomized Controlled Trial[Publication Type] OR Equivalence Trial[Publication Type] OR Pragmatic Clinical Trial[Publication Type] OR random*[All Fields] |
| #6 | #3 AND #4 AND #5 |

## Search strategy of Embase.

| No. | Search items |
| --- | --- |
| #1 | 'septic shock'/exp OR 'shock, septic':ti,ab,kw OR 'septic shock':ti,ab,kw OR 'shock, toxic':ti,ab,kw OR 'toxic shock syndrome':ti,ab,kw OR 'shock syndrome, toxic':ti,ab,kw OR 'toxic shock syndromes':ti,ab,kw OR 'toxic shock':ti,ab,kw OR 'shock, endotoxic':ti,ab,kw OR 'endotoxin shock':ti,ab,kw OR 'endotoxin shocks':ti,ab,kw OR 'shock, endotoxin':ti,ab,kw OR 'shocks, endotoxin':ti,ab,kw OR 'sepsis shock':ti,ab,kw OR 'infection shock':ti,ab,kw OR 'infectious shock':ti,ab,kw OR 'infective shock':ti,ab,kw |
| #2 | 'chinese herbal injection':ti,ab,kw OR 'chinese herbal injections':ti,ab,kw OR xingnaojing:ti,ab,kw OR danshen:ti,ab,kw OR shenqifuzheng:ti,ab,kw OR shuxuetong:ti,ab,kw OR huangqi:ti,ab,kw OR tanreqing:ti,ab,kw OR reduning:ti,ab,kw OR xiyanping:ti,ab,kw OR qingkailing:ti,ab,kw OR yiqifuzheng:ti,ab,kw OR yiqifumai:ti,ab,kw OR xuebijing:ti,ab,kw OR shenfu:ti,ab,kw OR shenmai:ti,ab,kw OR shengmai:ti,ab,kw |
| #3 | 'randomized controlled trial'/exp OR 'equivalence trial'/exp OR 'non-inferiority trial'/exp OR 'pragmatic trial'/exp OR 'superiority trial'/exp OR 'controlled clinical trial':it OR 'randomized controlled trial':it OR 'equivalence trial':it OR 'pragmatic clinical trial':it OR 'superiority trial':it OR 'non-inferiority trial':it OR random* |
| #4 | #1 AND #2 AND #3 |

## Search strategy of Cochrane Library.

| No. | Search items |
| --- | --- |
| #1 | MeSH descriptor: [Shock, Septic] explode all trees |
| #2 | (Shock, Septic):ti,ab,kw OR (Septic Shock):ti,ab,kw OR (Shock, Toxic):ti,ab,kw OR (Toxic Shock Syndrome):ti,ab,kw OR (Shock Syndrome, Toxic):ti,ab,kw OR (Toxic Shock Syndromes):ti,ab,kw OR (Toxic Shock):ti,ab,kw OR (Shock, Endotoxic):ti,ab,kw OR (Endotoxin Shock):ti,ab,kw OR (Endotoxin Shocks):ti,ab,kw OR (Shock, Endotoxin):ti,ab,kw OR (Shocks, Endotoxin):ti,ab,kw OR (Sepsis Shock):ti,ab,kw OR (Infection Shock):ti,ab,kw OR (Infectious Shock):ti,ab,kw OR (Infective Shock):ti,ab,kw |
| #3 | #1 OR #2 |
| #4 | (Chinese Herbal Injection):ti,ab,kw OR (Chinese Herbal Injections):ti,ab,kw OR (xingnaojing):ti,ab,kw OR (danshen):ti,ab,kw OR (shenqifuzheng):ti,ab,kw OR (shuxuetong):ti,ab,kw OR (huangqi):ti,ab,kw OR (tanreqing):ti,ab,kw OR (reduning):ti,ab,kw OR (xiyanping):ti,ab,kw OR (qingkailing):ti,ab,kw OR (yiqifuzheng):ti,ab,kw OR (yiqifumai):ti,ab,kw OR (xuebijing):ti,ab,kw OR (shenfu):ti,ab,kw OR (shenmai):ti,ab,kw OR (shengmai):ti,ab,kw |
| #5 | MeSH descriptor: [Randomized Controlled Trial] explode all trees |
| #6 | (Randomized Controlled Trial):pt OR (Controlled Clinical Trial):pt OR (Equivalence Trial):pt OR (Pragmatic Clinical Trial):pt OR (random*) |
| #7 | #5 OR #6 |
| #8 | #3 AND #4 AND #7 |

## Search strategy of Web of Science.

| No. | Search items |
| --- | --- |
| #1 | TS=(Shock, Septic) OR TS=(Septic Shock) OR TS=(Shock, Toxic) OR TS=(Toxic Shock Syndrome) OR TS=(Shock Syndrome, Toxic) OR TS=(Toxic Shock Syndromes) OR TS=(Toxic Shock) OR TS=(Shock, Endotoxic) OR TS=(Endotoxin Shock) OR TS=(Endotoxin Shocks) OR TS=(Shock, Endotoxin) OR TS=(Shocks, Endotoxin) OR TS=(Sepsis Shock) OR TS=(Infection Shock) OR TS=(Infectious Shock) OR TS=(Infective Shock) |
| #2 | TS=(Chinese Herbal Injection) OR TS=(Chinese Herbal Injections) OR TS=(yiqifuzheng) OR TS=(yiqifumai) OR TS=(xuebijing) OR TS=(shenfu) OR TS=(shenmai) OR TS=(shengmai) OR TS=(xingnaojing) OR TS=(danshen) OR TS=(huangqi) OR TS=(shenqifuzheng) OR TS=(shuxuetong) OR TS=(tanreqing) OR TS=(reduning) OR TS=(xiyanping) OR TS=(qingkailing) |
| #3 | #1 AND #2 |

# File 3: Included studies.

Cao, S. X., Huang, H., and Wang, J. (2021). Efficacy of xuebijing for inflammatory response in patients with sepsic shock. *Shenzhen Journal of Integrated Traditional Chinese and Western Medicine* 31 (09)**,** 42-44. doi:10.16458/j.cnki.1007-0893.2021.09.019

Chen, D. X. (2020). The efficacy and safety of xuebijing injection combined with ulinastatin for treating septic shock. *Diet and Health Care* 7 (19)**,** 65-66.

Chen, R. J., Guo, X., Wang, B., Cheng, B., and Yuqiang, G. (2017). Protective Effects of Shenfu Injection on Acute Kidney Injury of Patients with Septic Shock. *Journal of Emergency in Traditional Chinese Medicine* 26 (06)**,** 1072-1075. doi:10.3969/j.issn.1004-745X.2017.06.044

Chen, R. J., Zhang, M., Pan, L., Cheng, B., Gong, Y., Jin, S., et al. (2015). Effect of Shenfu Injection on Inflammatory Response and Immune Function in Patients with Septic Shock: a Randomized Controlled Study. *Chinese Archives of Traditional Chinese Medicine* 33 (10)**,** 2461-2464. doi:10.13193/j.issn.1673-7717.2015.10.048

Chen, S. (2018). Effects of xuebijing injection combined with levosimendan in septic shock. *Modern Journal of Integrated Traditional Chinese and Western Medicine* 27 (1). doi:10.3969/j.issn.1008-8849.2018.01

Chen, Z. (2016). Analysis of clinical effect in shenfu Iinjection in the treatment of septic shock. *Modern Regimen* (02)**,** 69. doi:CNKI:SUN:YSXD.0.2016-02-060

Cheng, T. C., Xu, Y., and Liang, W. (2018). Effect of Shenfu Injection as an adjuvant therapy in the treatment of patients with severe septic shock. *China Modern Medicine* 25 (34)**,** 44-47. doi:10.3969/j.issn.1674-4721.2018.34.015

Cui, L. C. (2021). Clinical efficacy and effect of inflammatory factors in shengmai injection combined with dopamine for septic shock. *Clinical Research* 29 (01)**,** 49-50.

Cui, Y., and Dai, L. (2016). Clinical efficacy and impact on hemodynamics in shenfu injection for septic shock. *Modern Journal of Integrated Traditional Chinese and Western Medicine* 25 (19)**,** 2120-2122. doi:10.3969/j.issn.1008-8849.2016.19.024

Dong, G. Y., and Shen, B. (2014). The role of Shenfu injection in the early cluster treatment of septic shock. *Journal of Emergency in Traditional Chinese Medicine* 23 (05)**,** 944-945. doi:10.3969/j.issn.1004-745X.2014.05.077

Fan, Y. X., Liu, W., Liu, C., Li, L., and Li, S. (2014). Clinical observation of Xuebijing in elderly septic shock. *Medical Aesthetics and Cosmetology* (11)**,** 124-124,125.

Gao, D. N. (2017). *Clinical Study on Shenqifuzheng, TCM Dialectical Types and Expression of Negative Costimulatory Molecules of Sepsis.* Doctor, Dalian Medical University.

Heng, J. F. (2013). Evaluation of the clinical efficacy in Xuebijing injection in the treatment of 32 cases of septic shock by blood lactic acid combined with APACHE Ⅱ score. *Journal of Emergency in Traditional Chinese Medicine* 22 (06)**,** 1047-1048. doi:10.3969/j.issn.1004-745X.2013.06.109

Huang, X. X., Xie, C., Meng, F., Liu, B., Wang, G., and Guo, Y. (2015). Effects of the Shenfu injection on septic shock by applying PiCCO evolution. *Clinical Journal of Chinese Medicine* 7 (25)**,** 52-53+55. doi:10.3969/j.issn.1674-7860.2015.25.027

Lai, Z. Z., Meng, J., Hu, M., Xu, X., and Li, C. (2018). Effects of Shenfu injection on hemodynamics and myocardial injury markers in patients combined with both septic shock and low cardiac output. *Zhejiang Journal of Integrated Traditional Chinese and Western Medicine* 28 (08)**,** 641-644. doi:10.3969/j.issn.1005-4561.2018.08.011

Lei, X. Y., and Li, Y. (2016). Effects of Shenfu Injection on Liver Function in Patients with Septic Shock in ICU. *Asia-Pacific Traditional Medicine* 12 (05)**,** 135-136. doi:10.11954/ytctyy.201605059

Li, C. L., and Chen, J. (2014). Application of Shenfu Injection on ICU Patients with Septic Shock. *Journal of Emergency in Traditional Chinese Medicine* 23 (4)**,** 608-609. doi:10.3969/j.issn.1004-745X.2014.04.018

Li, J. Y. (2012). *Clinical study on treatment of septic shock with Shenfu injection.* Master, Zhejiang University.

Li, J. S. (2020). *Clinical study on the effect of Shenfu injection on blood lactic acid level and curative effect in patients with septic shock.* Master, Guangzhou University of Chinese Medicine.

Li, L. W. (2017). *Effect of Xuebijing Injection on Blood Coagulation Function and Inflammatory Factors in Patients with Septic Shock.* Master, Hebei Medical University.

Li, M. Q., Pan, C. G., Wang, X. M., Mo, X., Shi, Z. X., Xu, J. Y., et al. (2015). Effect of the Shenfu Injection Combined with Early Goal-Directed Therapy on Organ Functions and Outcomes of Septic Shock Patients. *Cell Biochemistry and Biophysics* 72 (3)**,** 807-812. doi:10.1007/s12013-015-0537-4

Li, M. L., Pan, T., Lv, L., Zhang, W., Tan, R., Liu, Z., et al. (2019). Effect of traditional Chinese medicine syndrome differentiation and standard bundle therapy in patients with septic shock. *Chinese Critical Care Medicine* (07)**,** 852-856. doi:10.3760/cma.j.issn.2095-4352.2019.07.011

Li, Q. (2018). *Effect of Xuebijing on blood coagulation and vascular endothelial function related factors in patients with septic shock.* Master, Hebei Medical University.

Li, Y., Zhang, X., Lin, P., Qiu, H., Wei, J., Cao, Y., et al. (2016). Effects of Shenfu Injection in the Treatment of Septic Shock Patients: A Multicenter, Controlled, Randomized, Open-Label Trial. *Evid Based Complement Alternat Med* 2016**,** 2565169. doi:10.1155/2016/2565169

Lin, B., Jiang, L., and Guo, Y. (2014). Effects of Shengmai injection on hemodynamics of patients with infectious shock. *Acta Medicinae Sinica* 27 (01)**,** 35-37. doi:CNKI:SUN:GLYX.0.2014-01-013

Lin, B., Jiang, L., and Guo, Y. (2019). Effect of Xuebijing Injection on Myocardial Protection in Patients with Septic Shock. *Guangxi Journal of Traditional Chinese Medicine* 42 (01)**,** 9-11. doi:10.3969/j.issn.1003-0719.2019.01.003

Liu, H. (2009). Impact of astragalus combined with glutamine on the concentration of nitric oxide and diamine oxidase in the plasma of the patients with septic shock. *Chinese Journal of Critical Care Medicine* 29 (11)**,** 986-989. doi:10.3969/j.issn.1002-1949.2009.11.008

Liu, M. L., Ye, B., and Shi, X. (2017). Analysis of curative effect of Shenfu injection on patients with septic shock. *Journal of Imaging Research and Medical Applications* 1 (14)**,** 171-173. doi:10.3969/j.issn.2096-3807.2017.14.107

Liu, P. F., and Yang, T. (2018). The effect of Shenmai injection in adjuvant treatment of septic shock and its influence on patients' immune function. *Tianjin Pharmacy* 30 (4)**,** 33-35. doi:10.3969/j.issn.1006-5687.2018.04.012

Liu, W. R., Lu, Q., Ti, C., Wei, X., Yang, L., and Ma, Z. (2019a). Clinical study on Shenmai Injection combined with hydrocortisone in treatment of senile septic shock. *Drugs & Clinic* 34 (09)**,** 2739-2743.

Liu, W. R., Zhang, Q., and Ti, C. (2019b). Effect of Shenmai Injection Combined with Hemoperfusion on Myocardial Inhibition of Septic Shock Patients. *Smart Healthcare* 5 (3)**,** 161-162,164. doi:10.19335/j.cnki.2096-1219.2019.03.080

Lu, D., Yu, G., Lv, T., and Ying, L. (2017). Effect of Shenmai Injection with Hemoperfusion on Hemodynamics and Tissue Perfusion in Septic Shock. *Chinese General Practice* 20 (34)**,** 4322-4325+4330. doi:10.3969/j.issn.1007-9572.2017.34.021

Lu, P. J. (2014). Exploration of the therapeutic effect of Shenqi Fuzheng injection on patients with septic shock after surgery. *Contemporary Medicine Forum* (19)**,** 29-30. doi:CNKI:SUN:QYWA.0.2014-19-026

Luo, R. C., Yue, J., Zhang, H., and Hu, C. (2009). Effect of Xuebijing Injection on Circulatory Function and Tissue Oxygenation in Patients with Septic Shock. *Practical Preventive Medicine* 16 (03)**,** 860-863. doi:10.3969/j.issn.1006-3110.2009.03.096

Luo, Y., Xu, M., Gou, F., and Fan, Y. (2019). Effect of Shengmai injection combined with dopamine on clinical efficacy and inflammatory factors in patients with septic shock. *Diet Health* 6 (42)**,** 33.

Ma, J. S., and Yang, X. (2013). Effect of Shenfu injection on endothelial cell function in patients with septic shock. *Chinese Journal of Gerontology* 33 (13). doi:10.3969/j.issn.1005-9202.2013.13.134

Meng, Q. L., Zhang, P., Han, X., Wang, L., Wang, Y., Huang, C., et al. (2018). Effect of xiyanping injections on tumor necrosis factor alpha and serum procalcitonin in elderly patients of septic shock. *Journal of Clinical Emergency* 19 (12)**,** 844-846. doi:10.13201/j.issn.1009-5918.2018.12.009

Pan, Y., and Chen, X. (2020). Application Value of Shenfu Injection in the Treatment of Septic Shock. *Chinese and Foreign Medical Research* 18 (27). doi:10.14033/j.cnki.cfmr.2020.27.019

Peng, Z. L., Zhang, H., Xu, L., Fan, C., Zhou, R., and Ju, Q. (2021). Analysis of the effect of Shengmai injection in the treatment of septic shock. *Contemporary Medicine Forum* 19 (10)**,** 74-76. doi:10.3969/j.issn.2095-7629.2021.10.042

Ren, D. H., Li, G., Sun, J., li, Z., Shi, Z., Bao, Z., et al. (2015). Effects of Shenmai Injection on Hemodynamic Measurements for Patients with Septic Shock. *Chinese Archives of Traditional Chinese Medicine* 33 (11)**,** 2675-2677. doi:10.13193/j.issn.1673-7717.2015.11.034

Sang, Z. Z., Gao, J., Jia, C., and Li, Y. (2019). The therapeutic effect of hydrocortisone combined with Shenfu injection on septic shock. *The Journal of Practical Medicine* 35 (18)**,** 2895-2899. doi:10.3969/j.issn.1006-5725.2019.18.014

Shi, B. Z., Yang, M., Dong, Y., Dong, X., Wang, L., Li, Y., et al. (2019). Clinical effects of Shenmai Injection combined with routine treatment on septic shock patients. *Chinese Traditional Patent Medicine* 41 (04)**,** 804-808. doi:10.3969/j.issn.1001-1528.2019.04.018

Shi, Y. J., Wang, E., Liu, X., Zhang, B., and Zheng, H. (2021). Effect of Shenfu injection on cardiac diastolic function in patients with septic shock and its therapeutic effect. *Chinese Journal of New Clinical Medicine* 14 (5)**,** 484-487. doi:10.3969/j.issn.1674-3806.2021.05.14

Sun, R. Q., Liang, M., Yang, h., Liu, Q., Ma, N., Wei, D., et al. (2020). Effect of Xuebijing on inflammatory response and prognosis in patients with septic shock. *Chinese Critical Care Medicine* 32 (04)**,** 458-462. doi:10.3760/cma.j.cn121430-20200401-00333

Tang, Z. L., Tang, X., Zhou, T., Qin, B., and Zeng, G. (2015). Effect of Shuxuetong on plasma von Willebrand factor and prognosis of patients with septic shock. *Clinical Medicine of China* 31 (11)**,** 1006-1008. doi:10.3760/cma.j.issn.1008-6315.2015.11.014

Wang, D. S. (2011). Study on the effect of Danshen on serum CRP, NO and PCT in patients with septic shock. *China Health Industry* (14)**,** 38. doi:CNKI:SUN:WSCY.0.2011-Z5-029

Wang, J. Y., Qiao, M., Peng, Y., Peng, X., Wen, G., Liu, J., et al. (2015). Clinical observation of Shenfu injection on improving hemodynamics and tissue perfusion in septic shock (high excretion and low resistance syndrome). *Journal of Emergency in Traditional Chinese Medicine* 24 (10)**,** 1827-1828. doi:10.3969/j.issn.1004-745X.2015.10.048

Wang, L. (2018). Effect of Xuebijing injection combined with atuomulan on inflammatory response in patients with septic shock. *Zhejiang Medical Journal* 40 (6). doi:10.12056/j.issn.1006-2785.2018.40.6.2017-549

Wang, Z. C., and Liu, L. (2015). "Clinical Observation on Treatment of Septic Shock by Integrated Traditional Chinese and Western Medicine", in: *Proceedings of the 2015 Emergency Academic Annual Conference and TCM Symposium on Emergency and Critical Diseases.* (Zhengzhou).

Xiao, Y. C., Li, Y., Wang, G., and Guo, Y. (2017). Clinical Observation of Shenfu Injection in the Treatment of Septic Shock. *Journal of Emergency in Traditional Chinese Medicine* 26 (09)**,** 1665-1667. doi:10.3969/j.issn.1004-745X.2017.09.052

Xie, Q. (2016). Effect of Xuebijing Injection on Hemodynamics and Inflammatory Factors in Patients with Septic Shock. *Modern Journal of Integrated Traditional Chinese and Western Medicine* 25 (31)**,** 3459-3461. doi:10.3969/j.issn.1008-8849.2016.31.014

Xie, R. F. (2016). Effects of Early Goal-directed Therapy Combined with Yiqi Fumai Lyophilized Powder on P(cv-a)CO2 and ScvO2 in Patients with Septic Shock. *Chinese Journal of Integrated Traditional and Western Medicine* 36 (3)**,** 306-309. doi:10.7661/cjim.2016.03.0306

Xu, R. (2019). Efficiency observation of blood purification therapy combined with Shenfu Injection in treatment of patients with septic shock. *Journal of Clinical Medicine in Practice* 23 (15)**,** 9-13. doi:10.7619/jcmp.201915003

Yan, Z. J. (2018). Efficacy of Shenfu injection combined with antibacterial drugs on patients with septic shock in intensive care unit and its effect on hemodynamic parameters and levels of inflammatory factors. *Anti-Infection Pharmacy* 15 (5). doi:10.13493/j.issn.1672-7878.2018.05-036

Yang, Y. J. (2020). Clinical effect analysis of Shenfu injection combined with low-dose glucocorticoid in the treatment of septic shock. *Oriental Medicated Diet* (22)**,** 116.

Yao, S. (2015). Effect of Shenfu Injection on Systemic Circulation, Oxygen Metabolism and Prognosis of Patients with Septic Shock. *Shenzhen Journal of Integrated Traditional Chinese and Western Medicine* 25 (24)**,** 23-25. doi:10.16458/j.cnki.1007-0893.2015.24.011

Yin, X., Hu, Z., Zuo, T., He, J., and Guo, L. (2018). Effect of Removing Blood Stasis and Detoxification Method on Hemodynamic Disorders with Septic Shock. *Journal of New Chinese Medicine* 50 (04)**,** 69-72. doi:10.13457/j.cnki.jncm.2018.04.018

Zhang, J.J. (2014). Efficacy of Xuebijing injection on the septic shock and its impact on the Procalcitonin(PCT) and C-reactive protein (CRP). *Chinese Journal of Primary Medicine and Pharmacy* 21 (08)**,** 1172-1174. doi:10.3760/cma.j.issn.1008-6706.2014.08.021

Zhang, J. M. (2019). Observe the effectiveness of Shenfu injection in the treatment of septic shock. *Medical Diet and Health* (6)**,** 126-127.

Zhang, L. (2016). Analysis of the application value of Shenmai injection in the treatment of patients with septic shock. *Zhejiang Journal of Traditional Chinese Medicine* 51 (12)**,** 922-923. doi:10.3969/j.issn.0411-8421.2016.12.048

Zhang, R. M., and Wang, B. (2016). Application of Xuebijing injection combined with low-dose hydrocortisone based on bundle therapy in elderly patients with septic shock. *Journal of Tianjin Medical University* 22 (05)**,** 439-441+444. doi:CNKI:SUN:TJYK.0.2016-05-020

Zhang, S. Y. (2017). Clinical observation of Shenfu Injection to improve septic shock. *Drugs & Clinic* 32 (6)**,** 1034-1038. doi:10.7501/j.issn.1674-5515.2017.06.017

Zhang, W. M., and Wang, Y. (2017). The curative effect analysis of Shenmai injection in the treatment of patients with septic shock and its effect on cellular immune function. *International Medicine and Health Guidance News* 23 (20)**,** 3228-3231. doi:10.3760/cma.j.issn.1007-1245.2017.20.029

Zhang, Y. N. (2017). Clinical effect of Shenfu injection on patients with severe septic shock in ICU. *Jilin Journal of Traditional Chinese Medicine* 37 (10)**,** 993-997. doi:10.13463/j.cnki.jlzyy.2017.10.007

Zhang, Y. H., Deng, M., Su, Y., and Ma, M. (2016). Clinical Observation of Shenqi Fuzheng Injection on Septic Shock. *Journal of Emergency in Traditional Chinese Medicine* 25 (12)**,** 2324-2326. doi:10.3969/j.issn.1004-745X.2016.12.035

Zhang, Y., and Suo, H. (2018). Clinical observation of Yiqi Fumai(freeze-dried) for Injection combined with noradrenaline in treatment of septic shock. *Drugs & Clinic* 33 (09)**,** 2414-2418. doi:10.7501/j.issn.1674-5515.2018.09.057

Zhao, N., and Zhang, D. (2020). The value of Shenfu injection in improving the treatment of septic shock. *Strait Pharmaceutical Journal* 32 (8)**,** 117-119. doi:10.3969/j.issn.1006-3765.2020.08.041

Zhao, W. P., Yang, X., Shui, W., Zhang, B., and Gu, C. (2019). Analysis of the curative effect of Xuebijing combined with blood purification in the treatment of septic shock. *Shenzhen Journal of Integrated Traditional Chinese and Western Medicine* 29 (2)**,** 28-30. doi:10.16458/j.cnki.1007-0893.2019.02.014

Zheng, X. S., Qin, G., Dong, Z., and Feng, Y. (2013). The effect of Xuebijing injection on PCT and CRP in patients with septic shock. *China Practical Medical* 8 (17)**,** 135-137. doi:10.3969/j.issn.1673-7555.2013.17.100

Zheng, Y., and Pan, C. (2014). Effect of Shenfu Injection on Oxygen Metabolism in Patients with Infection Shock. *Chinese Archives of Traditional Chinese Medicine* 32 (11)**,** 2770-2772. doi:10.13193/j.issn.1673-7717.2014.11.063

Zhong, J. (2017). The effect of Xuebijing Injection on patients with septic shock in the clearance rate and SOFA score of lactic acid. *Journal of North Pharmacy* 14 (08)**,** 138-139. doi:10.3969/j.issn.1672-8351.2017.08.121

Zhong, J. X., Xu, C., and Peng, J. (2019). Clinical observation of continuous blood purification combined with Xuebijing injection in the treatment of patients with septic shock. *Internal Medicine of China* 14 (03)**,** 361-363. doi:10.16121/j.cnki.cn45-1347/r.2019.03.34

Zhong, K. L., and Jiang, B. (2015). Observation of therapeutic effects of shenfu Injection treating patients with septic shock. *Yi Yao Qian Yan* 5 (33)**,** 111-112. doi:10.3969/j.issn.2095-1752.2015.33.095

Zhou, C. L. (2014). *Clinical study on the effect of Shenfu injection on early volume resuscitation tissue perfusion in patients with septic shock.* Master, Shandong University Of Traditional Chinese Medicine.

Zhou, L. Q., and Wang, Y. (2016). Effect of Xuebijing injection on lactate clearance rate and the score of SOFA of septic shock patients. *Ji Lin Yi Xue* 37 (06)**,** 1339-1342. doi:10.3969/j.issn.1004-0412.2016.06.021

Zhou, L., Zhang, W., Chen, X., Lu, N., Jiang, Y., and Xie, J. (2013). Effects of Shenfu Injection on Tissue perfusion and Systemic Oxygen Metabolism in patients with Septic Shock. *Journal of Emergency in Traditional Chinese Medicine* 22 (09)**,** 1505-1507. doi:10.3969/j.issn.1004-745X.2013.09.022

Zou, H., Qian, H., and Zhao, H. (2020). The clinical effect of Shengmai injection combined with norepinephrine in the treatment of septic shock. *World Latest Medicine Information* 20 (31)**,** 147-148. doi:10.3969/j.issn.1671-3141.2020.31.090

# File 4: Detailed information on included CHIs.

| **Chinese herbal injection** | **Source** | **Species /Raw materials** | **Scientific name of the plant** | **Indication** | **Quality control reported. (Y/N)** | **Chemical analysis reported (Y/N)** |
| --- | --- | --- | --- | --- | --- | --- |
| Shenfu injection | Ya'an Sanjiu Pharmaceutical Co., Ltd. | Ginseng Radix et Rhizoma Rubra, Aconiti Lateralis Radix Praeparata | *Panax ginseng* C. A. Mey., *Aconitum carmichaelii* Debeaux. | Syndrome of Yang Qi Exhaustion (infectious, hemorrhagic shock, etc.); palpitation, cough, asthma, stomachache, diarrhea, or arthralgia caused by Yang Deficiency (Qi Deficiency) | Y-National Pharmaceutical Standard: Z51020664; National Food and Drug Administration National Drug Standard: WS3-B-3427-98-2013 | N |
| Shenmai injection | Ya'an Sanjiu Pharmaceutical Co., Ltd.;  Chiatai Qingchunbao Pharmaceutical Co., Ltd.;  Hebei Shenwei Pharmaceutical Co., Ltd.;  Sichuan University Huaxi Pharmaceutical Co., Ltd.;  Yunnan Gejiu Biopharmaceutical Co., Ltd.;  Sichuan Shenghe Pharmaceutical Co., Ltd.;  Dali Pharmaceutical Co., Ltd. | Ginseng Radix et Rhizoma Rubra, Radix Ophiopogonis | *Panax ginseng* C. A. Mey., *Ophiopogon japonicus* (Thunb.) Ker-Gawl. | Shock of deficiency of both Qi and Yin, coronary heart virus myocarditis, chronic cor pulmonale, and granulocytopenia can improve the immune function of cancer patients, enhance their immune function when combined with chemotherapeutic drugs, and reduce the toxic and side effects caused by chemotherapeutic drugs | Y-National Pharmaceutical Standard: Z51021845, Z33020019, Z13020889, Z51021353, Z53021720, Z51021263, Z20093649; National Food and Drug Administration National Drug Standard: WS3-B-3428-98-2010Z | N |
| Shengmai injection | Ya'an Sanjiu Pharmaceutical Co., Ltd.; Shanxi Taihang Pharmaceutical Co., Ltd.; Suzhong Pharmaceutical Group Co., Ltd.; Changshu Leiyunshang Pharmaceutical Co., Ltd.; Ji'an Yisheng Pharmaceutical Co., Ltd.; Wuliangye Group Yibin Pharmaceutical Co., Ltd.; Sichuan University Huaxi Pharmaceutical Co., Ltd.; Shanghai Hutchison Pharmaceutical Co., Ltd. | Ginseng Radix et Rhizoma Rubra, Radix Ophiopogonis, Schisandrae Chinensis Fructus | *Panax ginseng* C. A. Mey., *Ophiopogon japonicus* (Thunb.) Ker-Gawl., *Schisandra chinensis* (Turcz.) Baill. | Palpitation, shortness of breath, myocardial infarction, cardiogenic shock, or septic shock, all of which belong to Qi and Yin Deficiency | Y-National Pharmaceutical Standard: Z51021921, Z14020812, Z32021056, Z20044155, Z22025251, Z51022476, Z20044178, Z31020241; National Food and Drug Administration National Drug Standard: WS-3-B-2865-98-2011 | N |
| Xuebijing injection | Tianjin Chase Sun Pharmaceutical Co., Ltd. | Carthami Flos, Paeoniae Radix Rubra, Chuanxiong Rhizoma, Salviae Miltiorrhizae Radix et Rhizoma, Angelicae Sinensis Radix | *Carthamus tinctorius* L., *Paeonia lactiflora* Pall., *Conioselinum anthriscoides 'Chuanxiong'*, *Salvia miltiorrhiza* Bunge, *Angelica sinensis* (Oliv.) Diels | Systemic inflammatory response syndrome induced by infection; multiple organ dysfunction syndromes | Y-National Pharmaceutical Standard: Z20040033; National Food and Drug Administration National Drug Standard: YBZ01242004-2010Z-2012 | N |
| Yiqifumai injection | Tasly Zhijiao Pharmaceutical Co., Ltd. | Ginseng Radix et Rhizoma Rubra 0.5g, Radix Ophiopogonis 1.5g, Schisandrae Chinensis Fructus 0.75g | *Panax ginseng* C. A. Mey., *Ophiopogon japonicus* (Thunb.) Ker-Gawl., *Schisandra chinensis* (Turcz.) Baill. | Sweating, fatigue, pale patients, palpitation, shortness of breath, or chest pain, caused by deficiency of both Qi and Yin; exertional angina; left heart insufficiency | Y-National Pharmaceutical Standard: Z20060463; National Food and Drug Administration National Drug Standard: YBZ07062006-2009Z-2015 | N |
| Danshen injection | Chiatai Qingchunbao Pharmaceutical Co., Ltd.; Lei Yunshang Pharmaceutical Co., Ltd.; Hebei Shenwei Pharmaceutical Co., Ltd.; Sinopharm Yibin Pharmaceutical Co., Ltd.; Jiangsu Shenlong Pharmaceutical Co., Ltd.; Shanghai Zhongxi Pharmaceutical Co., Ltd. | Salviae Miltiorrhizae Radix et Rhizoma | *Salvia miltiorrhiza* Bunge | Promoting blood circulation, nourishing the heart; used for coronary heart diseases or circulatory disturbance | Y-National Pharmaceutical Standard: Z33020177; Z44022072; Z13020776; Z51020167; Z32020161; Z31020345; National Food and Drug Administration National Drug Standard: WS-3-B-3766-98-2011 | N |
| Huangqi injection | Zhejiang Jiuxu Pharmaceutical Co., Ltd.; Harbin Shengtai Biopharmaceutical Co., Ltd.; China Resources Double-Crane Pharmaceutical Co., Ltd.; Shanghai Xinya Pharmaceutical Gaoyou Co., Ltd.; Chiatai Qingchunbao Pharmaceutical Co., Ltd. | Astragali Radix 4g | *Astragalus mongholicus* Bunge | Tonifying Qi and warding off Evil; used for viral myocarditis, cardiac insufficiency, hepatitis, and weak state (including shock) | Y-National Pharmaceutical Standard: Z19993151; Z23020821; Z20054284; Z32021256; Z33020179; Approval for Revision of National Drug Standards of the State Drug Administration: 2001ZFBO171 | N |
| Xiyanping injection | Jiangxi Qingfeng Pharmaceutical Co., Ltd. | sulfonated Andrographolides | *Andrographis paniculata* (Burm. f.) Nees | Clearing heat and detoxifying; used for various infectious diseases. | Y-National Pharmaceutical Standard: Z20026249; National Food and Drug Administration National Drug Standard: WS-10863(ZD-0863)-2002-2011-Z | N |
| Shenqifuzheng injection | Livzon Pharmaceutical Group Co., Ltd. | Codonopsis Radix, Astragali Radix | *Codonopsis pilosula* (Franch.) Nannf., *Astragalus mongholicus* Bunge | Weak state (including shock); also used in the treatment of lung cancer and stomach cancer | Y-National Pharmaceutical Standard: Z19990065; National Food and Drug Administration National Drug Standard: YBZ05052017 | N |
| Shuxuetong injection | Livzon Pharmaceutical Group Co., Ltd. | Hirudo (animal drug), Pheretima (animal drug) | *Whitmania pigra* Whitman, *Pheretima aspergillum* (E. Perrier) | Acute cerebral infarction, circulatory disturbance | Y-National Pharmaceutical Standard: Z200010100; National Food and Drug Administration National Drug Standard: WS3-548(Z-048)-2005(Z) | N |

# File 5: Brooks-Gelman-Rubin plots.


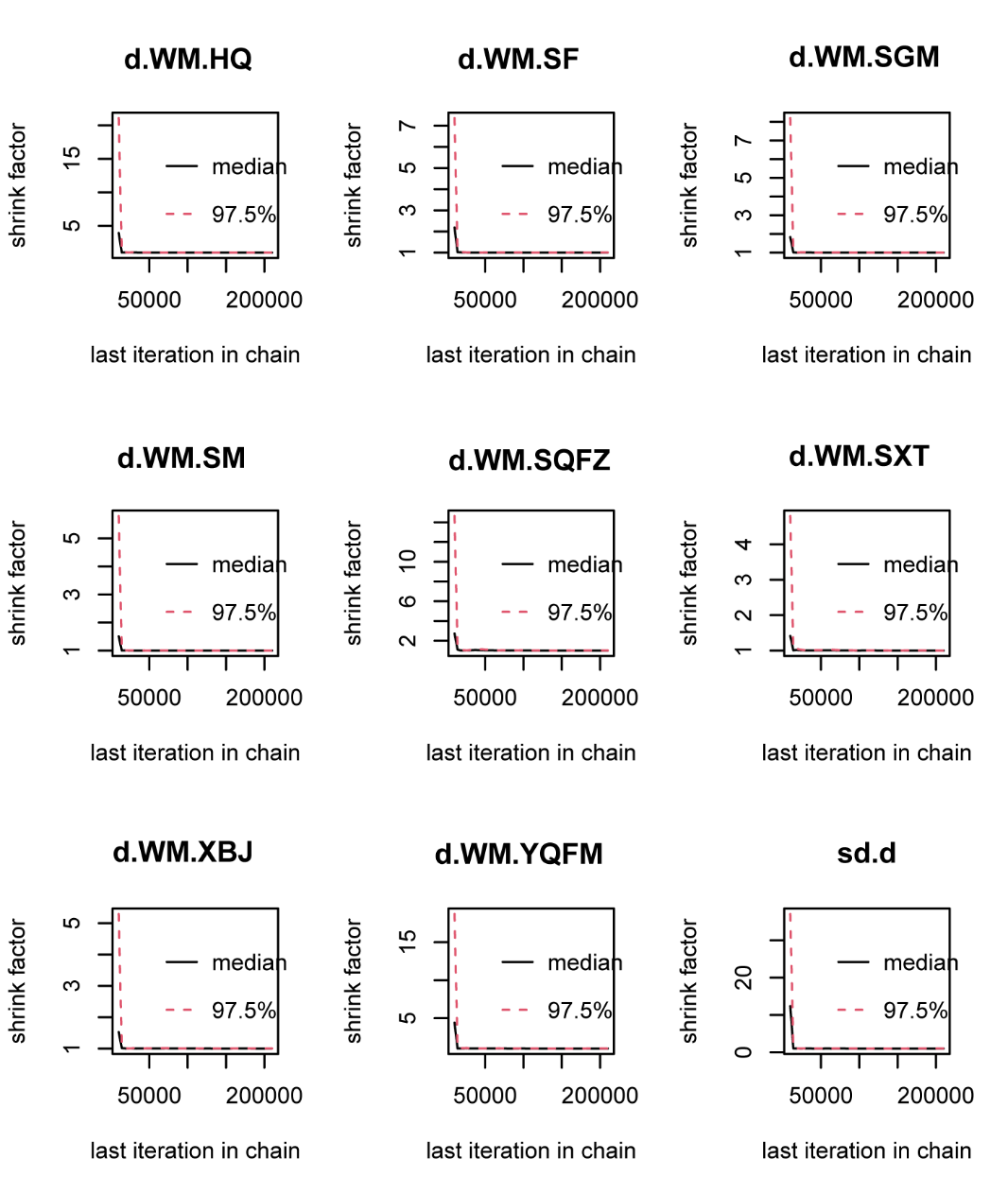


Supplementary Figure 1. Brooks-Gelman-Rubin plot for the efficacy of the comparison among different treatments for septic shock in 28-day-mortality. WM, Western Medicine; SF, Shenfu injection; SM, Shenmai injection; SGM, Shengmai injection; XBJ, Xuebijing injection; YQFM, Yiqifumai injection; HQ, Huangqi injection; SXT, Shuxuetong injection; SQFZ, Shenqifuzheng injection.


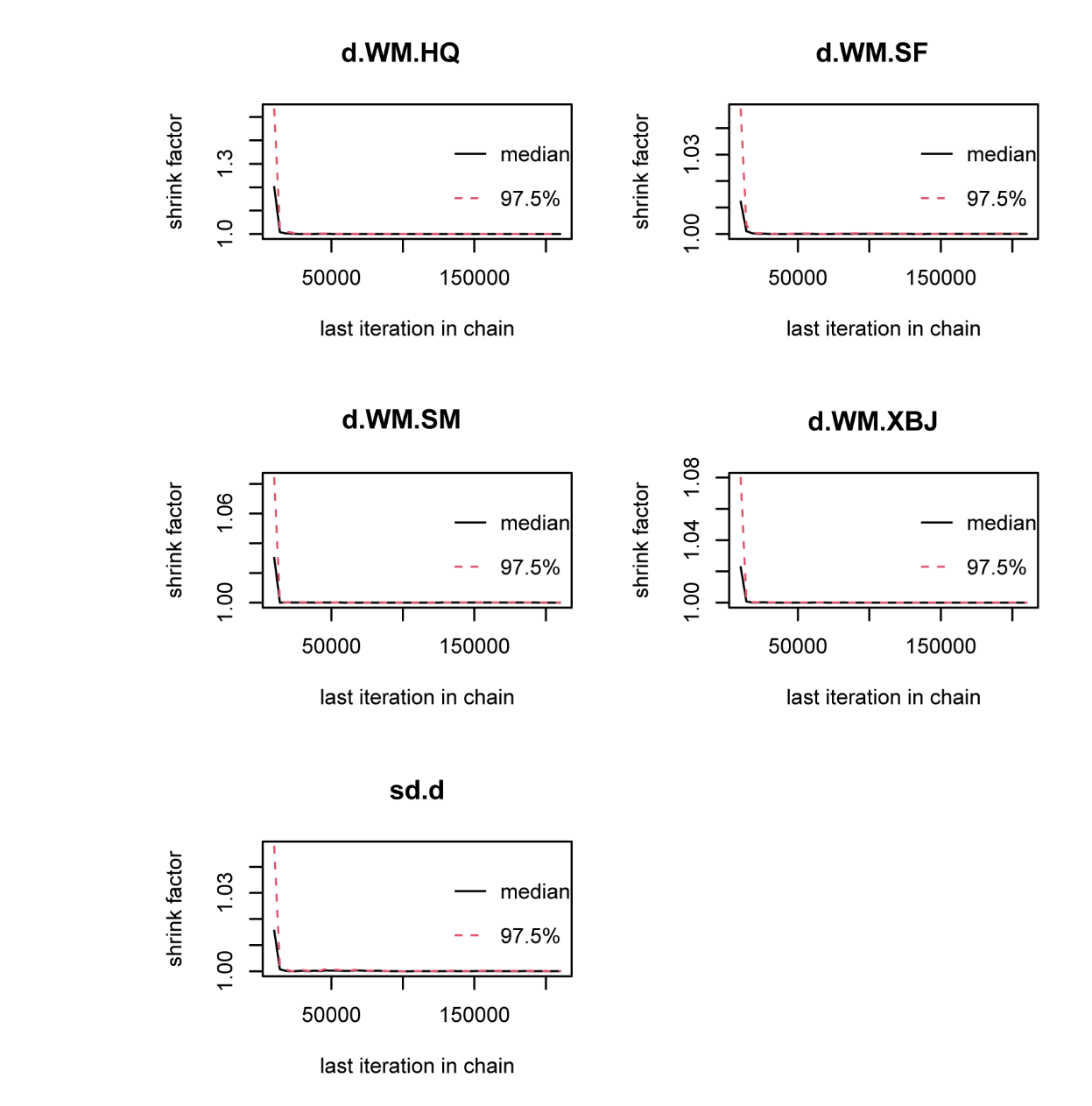


Supplementary Figure 2. Brooks-Gelman-Rubin plot for the efficacy of the comparison among different treatments for septic shock in ICU length of stay. WM, Western Medicine; SF, Shenfu injection; SM, Shenmai injection; XBJ, Xuebijing injection; HQ, Huangqi injection.


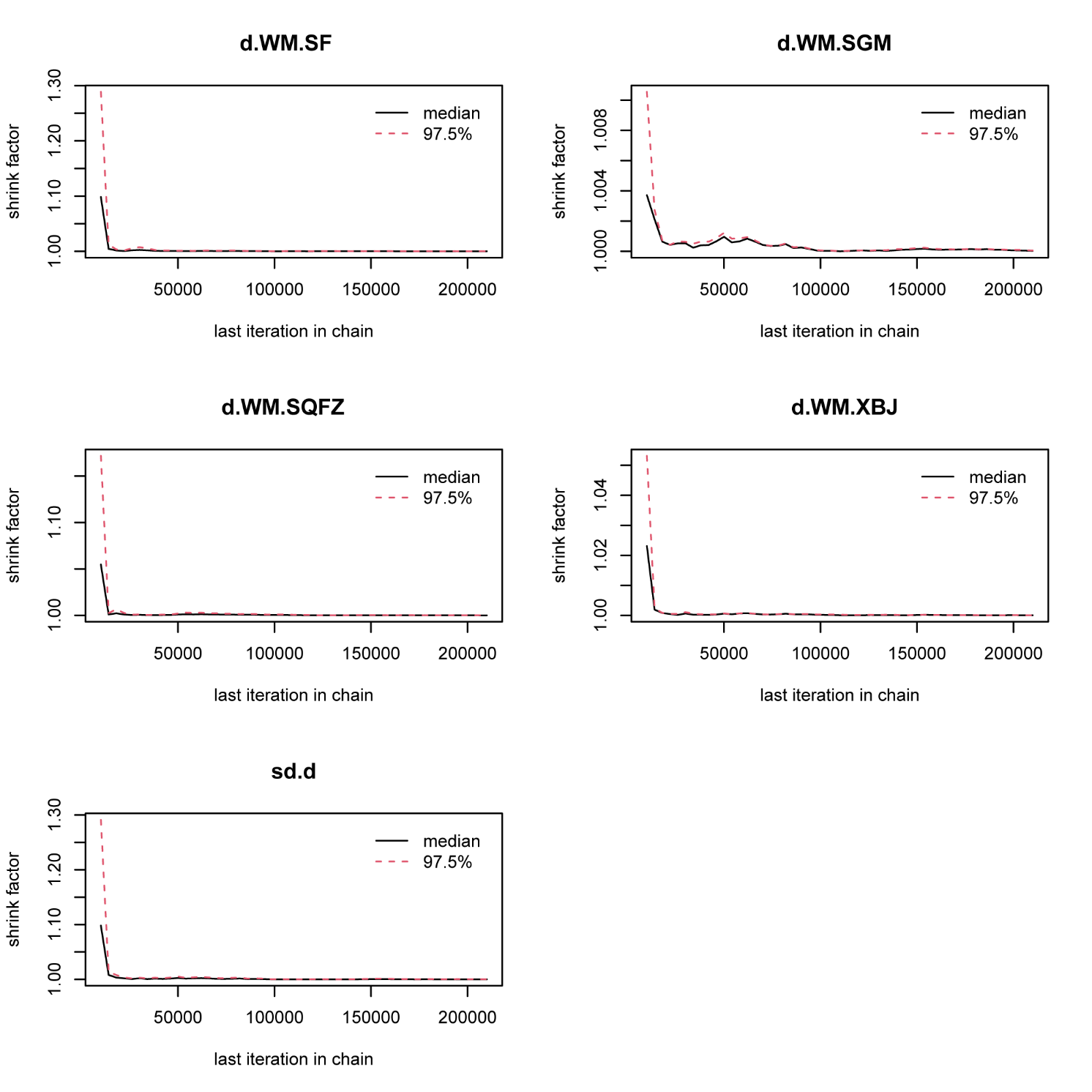


Supplementary Figure 3. Brooks-Gelman-Rubin plot for the efficacy of the comparison among different treatments for septic shock in hospital length of stay. WM, Western Medicine; SF, Shenfu injection; SGM, Shengmai injection; XBJ, Xuebijing injection; SQFZ, Shenqifuzheng injection.


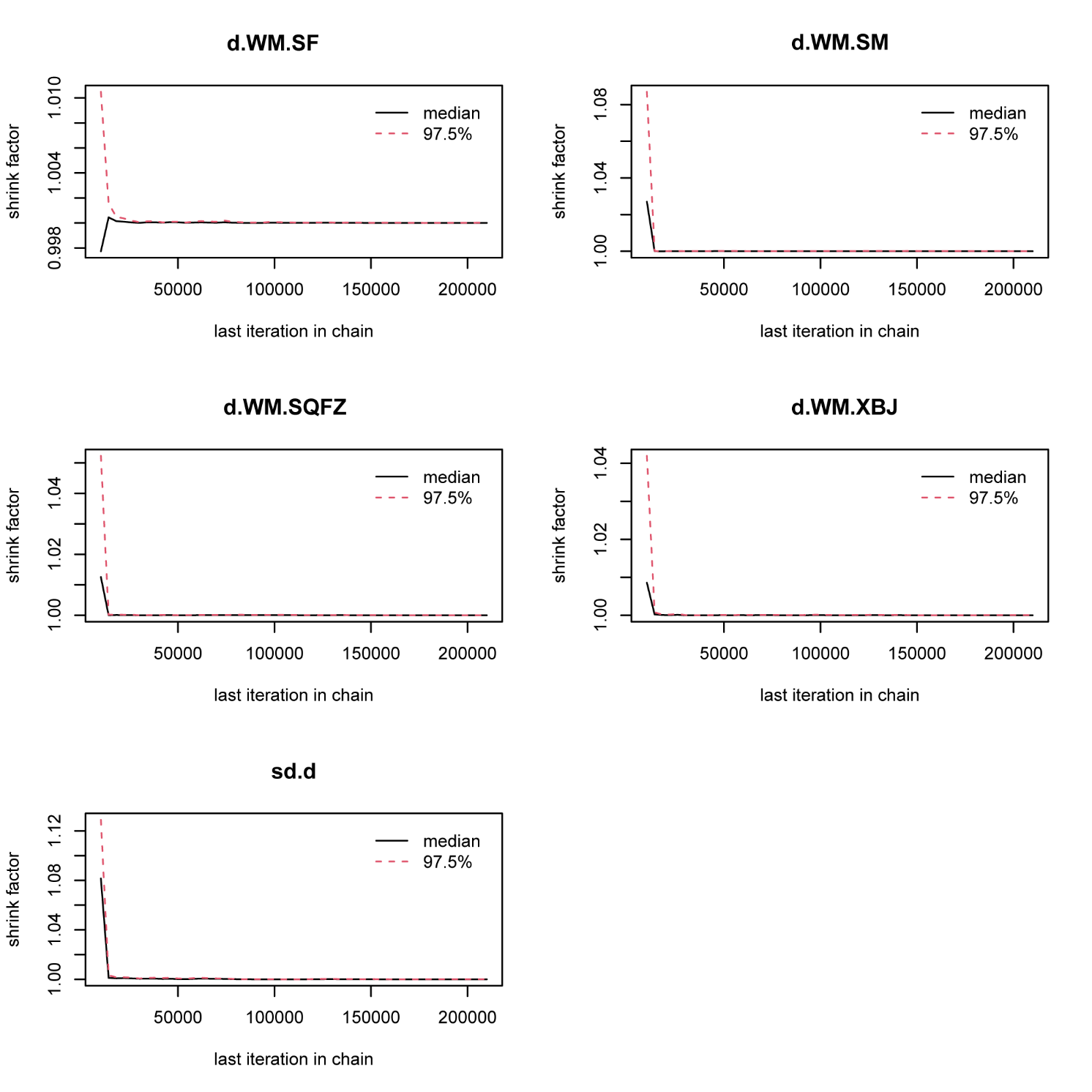


Supplementary Figure 4. Brooks-Gelman-Rubin plot for the efficacy of the comparison among different treatments for septic shock in SOFA score at day 7 after interventions. WM, Western Medicine; SF, Shenfu injection; SGM, Shengmai injection; XBJ, Xuebijing injection; SQFZ, Shenqifuzheng injection.


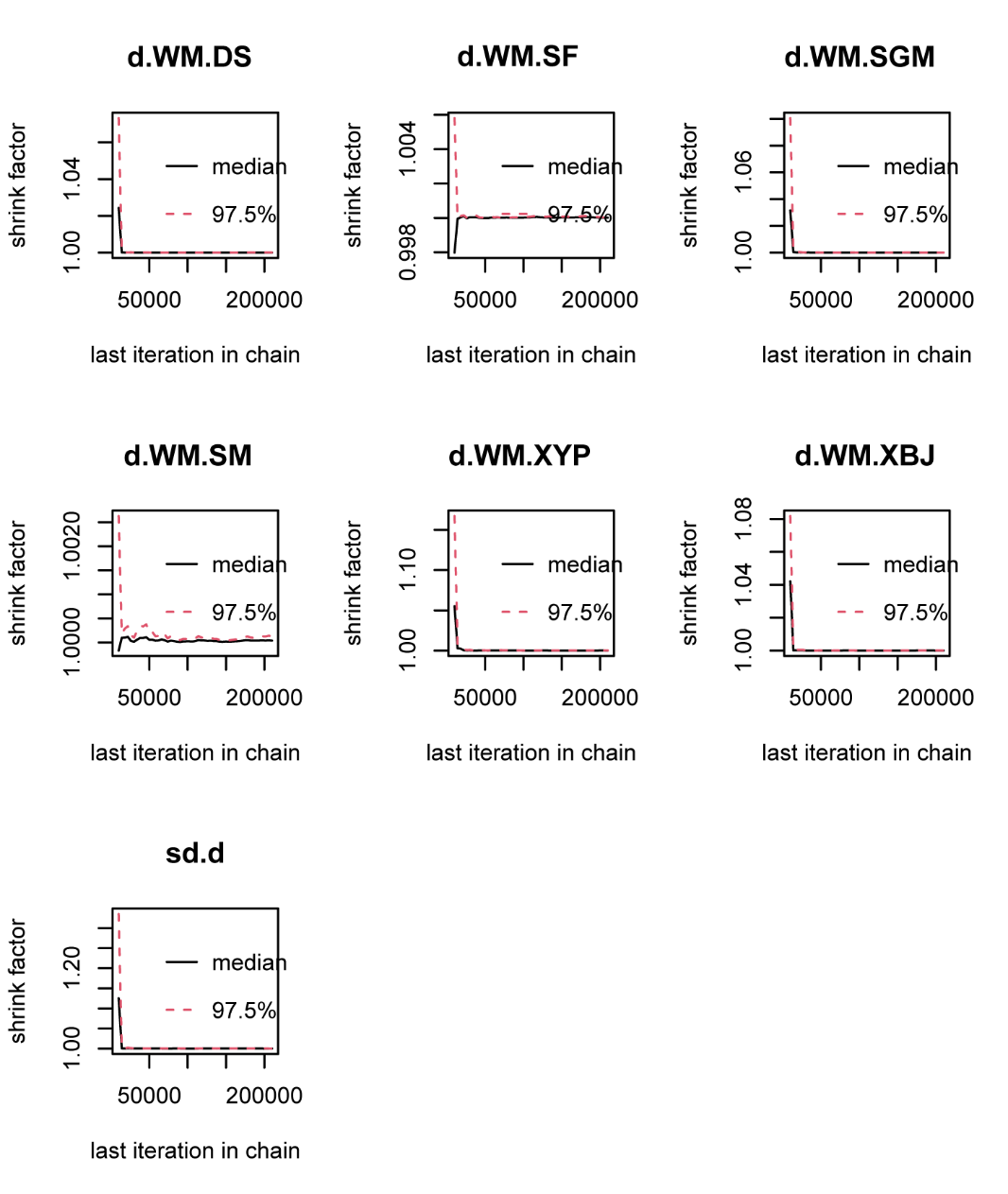


Supplementary Figure 5. Brooks-Gelman-Rubin plot for the efficacy of the comparison among different treatments for septic shock in procalcitonin level at day 7 after interventions. WM, Western Medicine; SF, Shenfu injection; SM, Shenmai injection; SGM, Shengmai injection; XBJ, Xuebijing injection; DS, Danshen injection; XYP, Xiyanping injection.


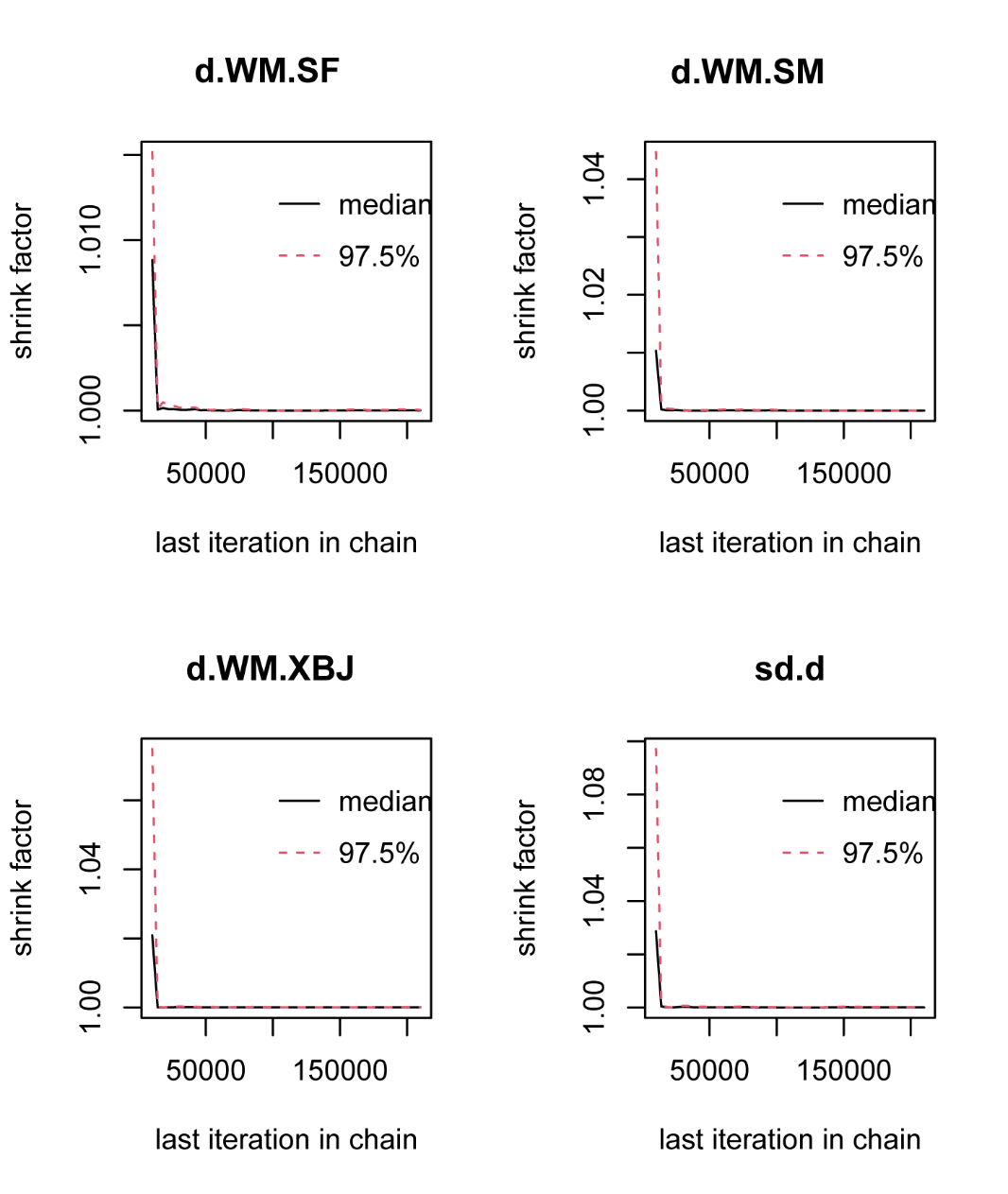


Supplementary Figure 6. Brooks-Gelman-Rubin plot for the efficacy of the comparison among different treatments for septic shock in serum lactate level at day 7 after interventions. WM, Western Medicine; SF, Shenfu injection; SM, Shenmai injection; XBJ, Xuebijing injection.

# File 6: Detailed information of heterogeneity analysis.

|  | 28-day-mortality (Global *I*^2^=0.0%) | ICU length of stay (Global *I*^2^=77.8%) | Hospital length of stay (Global *I*^2^=14.1%) | SOFA score at 7-day (Global *I*^2^=91.1%) | Procalcitonin level at 7-day (Global *I*^2^=99.6%) | Serum lactate level at 7-day (Global *I*^2^=93.5%) |
| --- | --- | --- | --- | --- | --- | --- |
| Pairwise comparison | Per-comparison *I*^2^ | Per-comparison *I*^2^ | Per-comparison *I*^2^ | Per-comparison *I*^2^ | Per-comparison *I*^2^ | Per-comparison *I*^2^ |
| SF+WM vs WM | 0.0% | 25.4% | 0.0% | 48.8% | 95.8% | 93.6% |
| SM+WM vs WM | 0.0% | - | - | - | 94.2% | 83.9% |
| SGM+WM vs WM | - | - | 71.5% | - | 74.3% | - |
| XBJ+WM vs WM | 37.2% | 96.2% | 33.2% | 92.9% | 86.9% | 84.8% |
| YQFM+WM vs WM | 0.0% | - | - | - | - | - |
| SQFZ+WM vs WM | 0.0% | - | - | - | - | - |

*Note: WM, Western Medicine; SF, Shenfu injection; SM, Shenmai injection; SGM, Shengmai injection; XBJ, Xuebijing injection; YQFM, Yiqifumai injection; SQFZ, Shenqifuzheng injection.*

# File 7: Forest plot of sensitivity analysis.


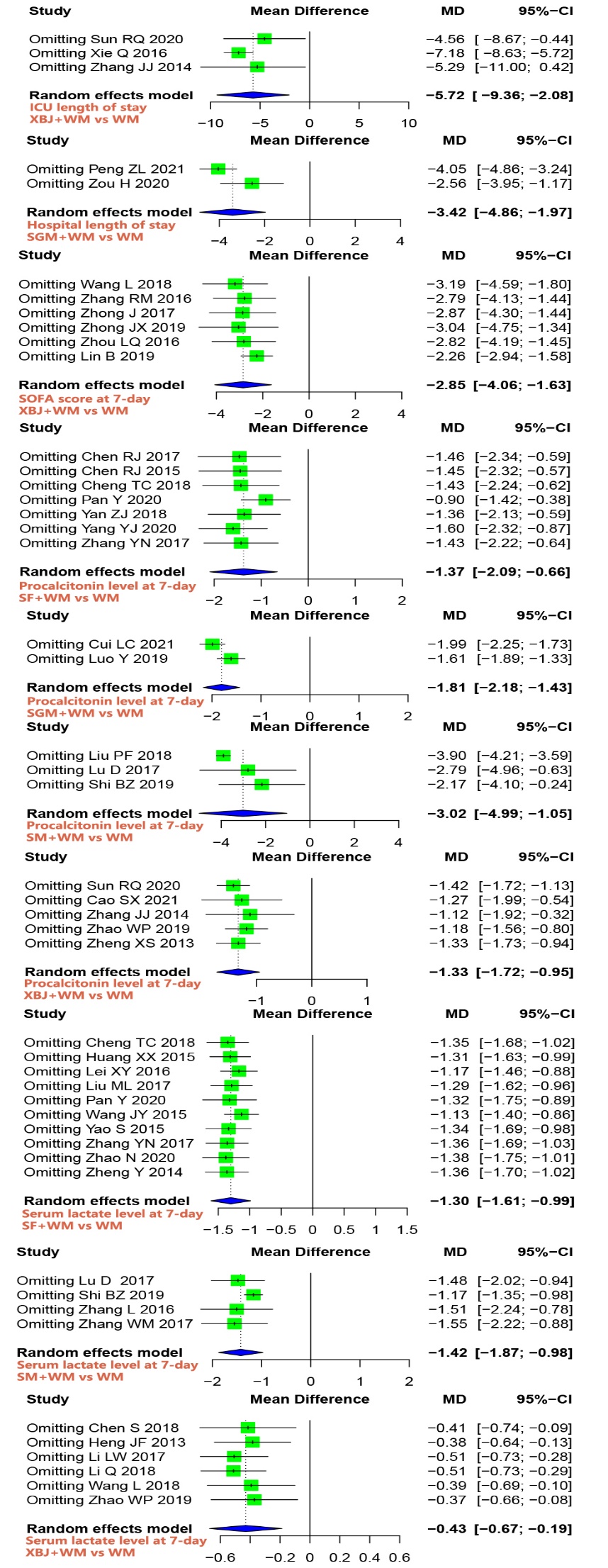


Supplementary Figure 7. Forest plot of sensitivity analysis. WM, Western Medicine; SF, Shenfu injection; SM, Shenmai injection; SGM, Shengmai injection; XBJ, Xuebijing injection.

# File 8: Subgroup analysis in diagnostic criteria according to Sepsis 2.0.


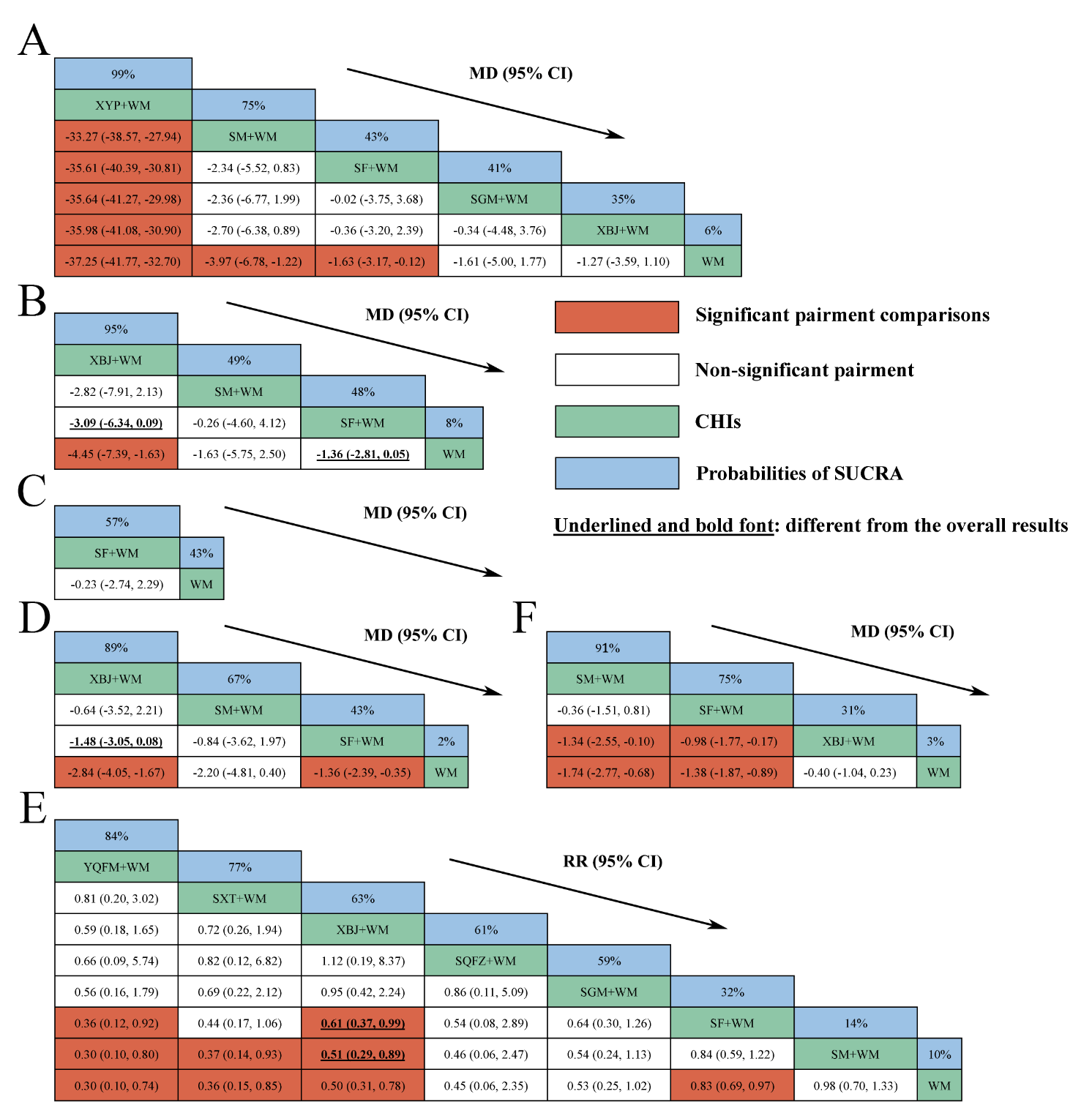


Supplementary Figure 8. **(A)** Procalcitonin level at day 7 after interventions; **(B)** ICU length of stay; **(C)** Hospital length of stay; **(D)** SOFA score at day 7 after interventions; **(E)** 28-day-motality; **(F)** Serum lactate level at day 7 after interventions; CHIs, Chinese herbal injections; WM, Western Medicine; SF, Shenfu injection; SM, Shenmai injection; SGM, Shengmai injection; XBJ, Xuebijing injection; YQFM, Yiqifumai injection; SXT, Shuxuetong injection; SQFZ, Shenqifuzheng injection; XYP, Xiyanping injection; SUCRA, surface under the cumulative ranking area curves. Highest probability of being the most efficient CHIs (With high SUCRA values) and Lowest probability of being the most efficient CHIs (With low SUCRA values).

# File 9: GRADE evaluation of the quality of evidence.

**9.1 Evaluation of the quality of evidence using GRADE framework for 28-day-motality.**

| **Comparison** | **Study limitation** | **Imprecision** | **Heterogeneity and inconsistency** | **Indirectness** | **Publication bias** | **Confidence in RR for 28-day-motality** |
| --- | --- | --- | --- | --- | --- | --- |
| YQFM+WM VS SXT+WM | 100% of the estimate from studies at “some concerns” (downgrade) | RR (95%CI): 0.84 (0.21, 3.14) (downgrade) | No head-to-head study and no heterogeneity.  There is no direct comparison between the interventions. | All the studies used the same diagnostic criterion for septic shock (Sepsis 2.0). | Comprehensive search strategy. | Low |
| YQFM+WM VS SQFZ+WM | 100% of the estimate from studies at “some concerns” (downgrade) | RR (95%CI): 0.86 (0.17, 4.95) (downgrade) | No head-to-head study and no heterogeneity.  There is no direct comparison between the interventions. | All the studies used the same diagnostic criterion for septic shock (Sepsis 2.0). | Comprehensive search strategy. | Low |
| YQFM+WM VS SGM+WM | 100% of the estimate from studies at “some concerns” (downgrade) | RR (95%CI): 0.57 (0.16, 1.86) (downgrade) | No head-to-head study and no heterogeneity.  There is no direct comparison between the interventions. | All the studies used the same diagnostic criterion for septic shock (Sepsis 2.0). | Comprehensive search strategy. | Low |
| YQFM+WM VS XBJ+WM | 100% of the estimate from studies at “some concerns” (downgrade) | RR (95%CI): 0.48 (0.15, 1.27) (downgrade) | No head-to-head study and no heterogeneity.  There is no direct comparison between the interventions. | The included studies used different diagnostic criteria for septic shock. (downgrade) | Comprehensive search strategy. | Very low |
| YQFM+WM VS HQ+WM | 100% of the estimate from studies at “some concerns” (downgrade) | RR (95%CI): 0.43 (0.10, 1.76) (downgrade) | No head-to-head study and no heterogeneity.  There is no direct comparison between the interventions. | The included studies used different diagnostic criteria for septic shock. (downgrade) | Comprehensive search strategy. | Very low |
| YQFM+WM VS SF+WM | 100% of the estimate from studies at “some concerns” (downgrade) | RR (95%CI): 0.38 (0.12, 0.96) | No head-to-head study and no heterogeneity.  There is no direct comparison between the interventions. | The included studies used different diagnostic criteria for septic shock. (downgrade) | Comprehensive search strategy. | Low |
| YQFM+WM VS SM+WM | 100% of the estimate from studies at “some concerns” (downgrade) | RR (95%CI): 0.31 (0.10, 0.82) | No head-to-head study and no heterogeneity.  There is no direct comparison between the interventions. | All the studies used the same diagnostic criterion for septic shock (Sepsis 2.0). | Comprehensive search strategy. | Moderate |
| YQFM+WM VS WM | 100% of the estimate from studies at “some concerns” (downgrade) | RR (95%CI): 0.31 (0.10, 0.76) | Mild heterogeneity according to *I*^2^=0.0%  There is no indirect comparison between the interventions. | All the studies used the same diagnostic criterion for septic shock (Sepsis 2.0). | Comprehensive search strategy. | Moderate |
| SXT+WM VS SQFZ+WM | 100% of the estimate from studies at “some concerns” (downgrade) | RR (95%CI): 1.03 (0.21, 5.61) (downgrade) | No head-to-head study and no heterogeneity.  There is no direct comparison between the interventions. | All the studies used the same diagnostic criterion for septic shock (Sepsis 2.0). | Comprehensive search strategy. | Low |
| SXT+WM VS SGM+WM | 100% of the estimate from studies at “some concerns” (downgrade) | RR (95%CI): 0.68 (0.22, 2.07) (downgrade) | No head-to-head study and no heterogeneity.  There is no direct comparison between the interventions. | All the studies used the same diagnostic criterion for septic shock (Sepsis 2.0). | Comprehensive search strategy. | Low |
| SXT+WM VS XBJ+WM | 100% of the estimate from studies at “some concerns” (downgrade) | RR (95%CI): 0.56 (0.21, 1.41) (downgrade) | No head-to-head study and no heterogeneity.  There is no direct comparison between the interventions. | The included studies used different diagnostic criteria for septic shock. (downgrade) | Comprehensive search strategy. | Very low |
| SXT+WM VS HQ+WM | 100% of the estimate from studies at “some concerns” (downgrade) | RR (95%CI): 0.51 (0.13, 2.03) (downgrade) | No head-to-head study and no heterogeneity.  There is no direct comparison between the interventions. | The included studies used different diagnostic criteria for septic shock. (downgrade) | Comprehensive search strategy. | Very low |
| SXT+WM VS SF+WM | 100% of the estimate from studies at “some concerns” (downgrade) | RR (95%CI): 0.45 (0.18, 1.07) (downgrade) | No head-to-head study and no heterogeneity.  There is no direct comparison between the interventions. | The included studies used different diagnostic criteria for septic shock. (downgrade) | Comprehensive search strategy. | Very low |
| SXT+WM VS SM+WM | 100% of the estimate from studies at “some concerns” (downgrade) | RR (95%CI): 0.37 (0.14, 0.91) | No head-to-head study and no heterogeneity.  There is no direct comparison between the interventions. | All the studies used the same diagnostic criterion for septic shock (Sepsis 2.0). | Comprehensive search strategy. | Moderate |
| SXT+WM VS WM | 100% of the estimate from studies at “some concerns” (downgrade) | RR (95%CI): 0.36 (0.14, 0.84) | No head-to-head studies of 2 or more and no heterogeneity.  There is no indirect comparison between the interventions. | All the studies used the same diagnostic criterion for septic shock (Sepsis 2.0). | Comprehensive search strategy. | Moderate |
| SQFZ+WM VS SGM+WM | 100% of the estimate from studies at “some concerns” (downgrade) | RR (95%CI): 0.66 (0.13, 2.88) (downgrade) | No head-to-head study and no heterogeneity.  There is no direct comparison between the interventions. | All the studies used the same diagnostic criterion for septic shock (Sepsis 2.0). | Comprehensive search strategy. | Low |
| SQFZ+WM VS XBJ+WM | 100% of the estimate from studies at “some concerns” (downgrade) | RR (95%CI): 0.55 (0.12, 2.06) (downgrade) | No head-to-head study and no heterogeneity.  There is no direct comparison between the interventions. | The included studies used different diagnostic criteria for septic shock. (downgrade) | Comprehensive search strategy. | Very low |
| SQFZ+WM VS HQ+WM | 100% of the estimate from studies at “some concerns” (downgrade) | RR (95%CI): 0.49 (0.08, 2.66) (downgrade) | No head-to-head study and no heterogeneity.  There is no direct comparison between the interventions. | The included studies used different diagnostic criteria for septic shock. (downgrade) | Comprehensive search strategy. | Very low |
| SQFZ+WM VS SF+WM | 100% of the estimate from studies at “some concerns” (downgrade) | RR (95%CI): 0.44 (0.10, 1.57) (downgrade) | No head-to-head study and no heterogeneity.  There is no direct comparison between the interventions. | The included studies used different diagnostic criteria for septic shock. (downgrade) | Comprehensive search strategy. | Very low |
| SQFZ+WM VS SM+WM | 100% of the estimate from studies at “some concerns” (downgrade) | RR (95%CI): 0.36 (0.08, 1.33) (downgrade) | No head-to-head study and no heterogeneity.  There is no direct comparison between the interventions. | All the studies used the same diagnostic criterion for septic shock (Sepsis 2.0). | Comprehensive search strategy. | Low |
| SQFZ+WM VS WM | 100% of the estimate from studies at “some concerns” (downgrade) | RR (95%CI): 0.35 (0.08, 1.24) (downgrade) | Mild heterogeneity according to *I*^2^=0.0%  There is no indirect comparison between the interventions. | All the studies used the same diagnostic criterion for septic shock (Sepsis 2.0). | Comprehensive search strategy. | Low |
| SGM+WM VS XBJ+WM | 100% of the estimate from studies at “some concerns” (downgrade) | RR (95%CI): 0.83 (0.36, 1.75) (downgrade) | No head-to-head study and no heterogeneity.  There is no direct comparison between the interventions. | The included studies used different diagnostic criteria for septic shock. (downgrade) | Comprehensive search strategy. | Very low |
| SGM+WM VS HQ+WM | 100% of the estimate from studies at “some concerns” (downgrade) | RR (95%CI): 0.75 (0.21, 2.74) (downgrade) | No head-to-head study and no heterogeneity.  There is no direct comparison between the interventions. | The included studies used different diagnostic criteria for septic shock. (downgrade) | Comprehensive search strategy. | Very low |
| SGM+WM VS SF+WM | 100% of the estimate from studies at “some concerns” (downgrade) | RR (95%CI): 0.67 (0.32, 1.30) (downgrade) | No head-to-head study and no heterogeneity.  There is no direct comparison between the interventions. | The included studies used different diagnostic criteria for septic shock. (downgrade) | Comprehensive search strategy. | Very low |
| SGM+WM VS SM+WM | 100% of the estimate from studies at “some concerns” (downgrade) | RR (95%CI): 0.55 (0.25, 1.11) (downgrade) | No head-to-head study and no heterogeneity.  There is no direct comparison between the interventions. | All the studies used the same diagnostic criterion for septic shock (Sepsis 2.0). | Comprehensive search strategy. | Low |
| SGM+WM VS WM | 100% of the estimate from studies at “some concerns” (downgrade) | RR (95%CI): 0.54 (0.25, 1.01) (downgrade) | No head-to-head studies of 2 or more and no heterogeneity.  There is no indirect comparison between the interventions. | All the studies used the same diagnostic criterion for septic shock (Sepsis 2.0). | Comprehensive search strategy. | Low |
| XBJ+WM VS HQ+WM | 100% of the estimate from studies at “some concerns” (downgrade) | RR (95%CI): 0.90 (0.30, 2.93) (downgrade) | No head-to-head study and no heterogeneity.  There is no direct comparison between the interventions. | The included studies used different diagnostic criteria for septic shock. (downgrade) | Comprehensive search strategy. | Very low |
| XBJ+WM VS SF+WM | 100% of the estimate from studies at “some concerns” (downgrade) | RR (95%CI): 0.81 (0.54, 1.19) (downgrade) | No head-to-head study and no heterogeneity.  There is no direct comparison between the interventions. | The included studies used different diagnostic criteria for septic shock. (downgrade) | Comprehensive search strategy. | Very low |
| XBJ+WM VS SM+WM | 100% of the estimate from studies at “some concerns” (downgrade) | RR (95%CI): 0.66 (0.40, 1.07) (downgrade) | No head-to-head study and no heterogeneity.  There is no direct comparison between the interventions. | The included studies used different diagnostic criteria for septic shock. (downgrade) | Comprehensive search strategy. | Very low |
| XBJ+WM VS WM | 100% of the estimate from studies at “some concerns” (downgrade) | RR (95%CI): 0.64 (0.44, 0.92) | Mild heterogeneity according to *I*^2^=37.2%  There is no indirect comparison between the interventions. | The included studies used different diagnostic criteria for septic shock. (downgrade) | Comprehensive search strategy. | Low |
| HQ+WM VS SF+WM | 100% of the estimate from studies at “some concerns” (downgrade) | RR (95%CI): 0.90 (0.29, 2.54) (downgrade) | No head-to-head study and no heterogeneity.  There is no direct comparison between the interventions. | The included studies used different diagnostic criteria for septic shock. (downgrade) | Comprehensive search strategy. | Very low |
| HQ+WM VS SM+WM | 100% of the estimate from studies at “some concerns” (downgrade) | RR (95%CI): 0.73 (0.23, 2.16) (downgrade) | No head-to-head study and no heterogeneity.  There is no direct comparison between the interventions. | The included studies used different diagnostic criteria for septic shock. (downgrade) | Comprehensive search strategy. | Very low |
| HQ+WM VS WM | 100% of the estimate from studies at “some concerns” (downgrade) | RR (95%CI): 0.72 (0.23, 1.99) (downgrade) | No head-to-head studies of 2 or more and no heterogeneity.  There is no indirect comparison between the interventions. | All the studies used the same diagnostic criterion for septic shock (Sepsis 1.0). | Comprehensive search strategy. | Low |
| SF+WM VS SM+WM | 100% of the estimate from studies at “some concerns” (downgrade) | RR (95%CI): 0.82 (0.58, 1.15) (downgrade) | No head-to-head study and no heterogeneity.  There is no direct comparison between the interventions. | The included studies used different diagnostic criteria for septic shock. (downgrade) | Comprehensive search strategy. | Very low |
| SF+WM VS WM | 100% of the estimate from studies at “some concerns” (downgrade) | RR (95%CI): 0.80 (0.69, 0.91) | Mild heterogeneity according to *I*^2^=0.0%  There is no indirect comparison between the interventions. | The included studies used different diagnostic criteria for septic shock. (downgrade) | Comprehensive search strategy. | Low |
| SM+WM VS WM | 100% of the estimate from studies at “some concerns” (downgrade) | RR (95%CI): 0.98 (0.71, 1.33) (downgrade) | Mild heterogeneity according to *I*^2^=0.0%  There is no indirect comparison between the interventions. | The included studies used different diagnostic criteria for septic shock. (downgrade) | Comprehensive search strategy. | Very low |
| Ranking of treatment | 100% of the estimate from studies at “some concerns” (downgrade) | Ranking probability of SUCRA suggested precision in a ranking of treatments. | Mild heterogeneity in network meta-analyses according to global *I*^2^ (0.0%).  There is no closed loop among the interventions. | The included studies used different diagnostic criteria for septic shock. (downgrade) | The funnel chart shows no obvious publication bias. | Low |

*Note: WM, Western Medicine; SF, Shenfu injection; SM, Shenmai injection; SGM, Shengmai injection; XBJ, Xuebijing injection; YQFM, Yiqifumai injection; HQ, Huangqi injection; SXT, Shuxuetong injection; SQFZ, Shenqifuzheng injection; SUCRA, surface under the cumulative ranking area curves.*

**9.2 Evaluation of the quality of evidence using GRADE framework for ICU length of stay.**

| **Comparison** | **Study limitation** | **Imprecision** | **Heterogeneity and inconsistency** | **Indirectness** | **Publication bias** | **Confidence in MD for ICU length of stay** |
| --- | --- | --- | --- | --- | --- | --- |
| XBJ+WM VS SF+WM | 100% of the estimate from studies at “some concerns” (downgrade) | MD (95%CI): -3.92 (-6.71, -1.36) | No head-to-head study and no heterogeneity.  There is no direct comparison between the interventions. | The included studies used different diagnostic criteria for septic shock. (downgrade) | Comprehensive search strategy. | Low |
| XBJ+WM VS SM+WM | 100% of the estimate from studies at “some concerns” (downgrade) | MD (95%CI): -3.80 (-8.53, 0.68) (downgrade) | No head-to-head study and no heterogeneity.  There is no direct comparison between the interventions. | The included studies used different diagnostic criteria for septic shock. (downgrade) | Comprehensive search strategy. | Very low |
| XBJ+WM VS HQ+WM | 100% of the estimate from studies at “some concerns” (downgrade) | MD (95%CI): -4.05 (-13.79, 5.65) (downgrade) | No head-to-head study and no heterogeneity.  There is no direct comparison between the interventions. | The included studies used different diagnostic criteria for septic shock. (downgrade) | Comprehensive search strategy. | Very low |
| XBJ+WM VS WM | 100% of the estimate from studies at “some concerns” (downgrade) | MD (95%CI): -5.44 (-7.92, -3.20) | Severe heterogeneity according to *I*^2^=96.2% (downgrade)  There is no indirect comparison between the interventions. | The included studies used different diagnostic criteria for septic shock. (downgrade) | Comprehensive search strategy. | Very low |
| SF+WM VS SM+WM | 100% of the estimate from studies at “some concerns” (downgrade) | MD (95%CI): -0.11 (-4.03, 4.25) (downgrade) | No head-to-head study and no heterogeneity.  There is no direct comparison between the interventions. | All the studies used the same diagnostic criterion for septic shock (Sepsis 2.0). | Comprehensive search strategy. | Low |
| SF+WM VS HQ+WM | 100% of the estimate from studies at “some concerns” (downgrade) | MD (95%CI): -0.11 (-9.42, 9.63) (downgrade) | No head-to-head study and no heterogeneity.  There is no direct comparison between the interventions. | The included studies used different diagnostic criteria for septic shock. (downgrade) | Comprehensive search strategy. | Very low |
| SF+WM VS WM | 100% of the estimate from studies at “some concerns” (downgrade) | MD (95%CI): -1.52 (-2.78, -0.27) | Mild heterogeneity according to *I*^2^=25.4%  There is no indirect comparison between the interventions. | All the studies used the same diagnostic criterion for septic shock (Sepsis 2.0). | Comprehensive search strategy. | Moderate |
| SM+WM VS HQ+WM | 100% of the estimate from studies at “some concerns” (downgrade) | MD (95%CI): -0.22 (-10.45, 10.03) (downgrade) | No head-to-head study and no heterogeneity.  There is no direct comparison between the interventions. | The included studies used different diagnostic criteria for septic shock. (downgrade) | Comprehensive search strategy. | Very low |
| SM+WM VS WM | 100% of the estimate from studies at “some concerns” (downgrade) | MD (95%CI): -1.64 (-5.59, 2.31) (downgrade) | No head-to-head studies of 2 or more and no heterogeneity.  There is no indirect comparison between the interventions. | All the studies used the same diagnostic criterion for septic shock (Sepsis 2.0). | Comprehensive search strategy. | Low |
| HQ+WM VS WM | 100% of the estimate from studies at “some concerns” (downgrade) | MD (95%CI): -1.42 (-10.87, 8.02) (downgrade) | No head-to-head studies of 2 or more and no heterogeneity.  There is no indirect comparison between the interventions. | All the studies used the same diagnostic criterion for septic shock (Sepsis 1.0). | Comprehensive search strategy. | Low |
| Ranking of treatment | 100% of the estimate from studies at “some concerns” (downgrade) | Ranking probability of SUCRA suggested precision in a ranking of treatments. | Severe heterogeneity in network meta-analyses according to global *I*^2^=77.8%. (downgrade)  There is no closed loop among the interventions. | The included studies used different diagnostic criteria for septic shock. (downgrade) | The funnel chart shows no obvious publication bias. | Very low |

*Note: WM, Western Medicine; SF, Shenfu injection; SM, Shenmai injection; HQ, Huangqi injection; XBJ, Xuebijing injection; SUCRA, surface under the cumulative ranking area curves.*

**9.3 Evaluation of the quality of evidence using GRADE framework for hospital length of stay.**

| **Comparison** | **Study limitation** | **Imprecision** | **Heterogeneity and inconsistency** | **Indirectness** | **Publication bias** | **Confidence in MD for hospital length of stay** |
| --- | --- | --- | --- | --- | --- | --- |
| SQFZ+WM VS SGM+WM | 100% of the estimate from studies at “some concerns” (downgrade) | MD (95%CI): -2.66 (-6.97, 1.44) (downgrade) | No head-to-head study and no heterogeneity.  There is no direct comparison between the interventions. | No report on which diagnostic criteria for septic shock were used. (downgrade) | Comprehensive search strategy. | Very low |
| SQFZ+WM VS XBJ+WM | 100% of the estimate from studies at “some concerns” (downgrade) | MD (95%CI): -3.04 (-7.06, 1.14) (downgrade) | No head-to-head study and no heterogeneity.  There is no direct comparison between the interventions. | All the studies used the same diagnostic criterion for septic shock (Sepsis 3.0). | Comprehensive search strategy. | Low |
| SQFZ+WM VS SF+WM | 100% of the estimate from studies at “some concerns” (downgrade) | MD (95%CI): -5.99 (-10.47, -1.57) | No head-to-head study and no heterogeneity.  There is no direct comparison between the interventions. | The included studies used different diagnostic criteria for septic shock. (downgrade) | Comprehensive search strategy. | Low |
| SQFZ+WM VS WM | 100% of the estimate from studies at “some concerns” (downgrade) | MD (95%CI): -6.15 (-9.89, -2.43) | No head-to-head studies of 2 or more and no heterogeneity.  There is no indirect comparison between the interventions. | No report on which diagnostic criteria for septic shock were used. (downgrade) | Comprehensive search strategy. | Low |
| SGM+WM VS XBJ+WM | 100% of the estimate from studies at “some concerns” (downgrade) | MD (95%CI): -0.41 (-2.78, 2.56) (downgrade) | No head-to-head study and no heterogeneity.  There is no direct comparison between the interventions. | All the studies used the same diagnostic criterion for septic shock (Sepsis 3.0). | Comprehensive search strategy. | Low |
| SGM+WM VS SF+WM | 100% of the estimate from studies at “some concerns” (downgrade) | MD (95%CI): -3.32 (-6.36, -0.15) | No head-to-head study and no heterogeneity.  There is no direct comparison between the interventions. | The included studies used different diagnostic criteria for septic shock. (downgrade) | Comprehensive search strategy. | Low |
| SGM+WM VS WM | 100% of the estimate from studies at “some concerns” (downgrade) | MD (95%CI): -3.51 (-5.33, -1.35) | Moderate heterogeneity according to *I*^2^=71.5%. (downgrade)  There is no indirect comparison between the interventions. | No report on which diagnostic criteria for septic shock were used. (downgrade) | Comprehensive search strategy. | Very low |
| XBJ+WM VS SF+WM | 100% of the estimate from studies at “some concerns” (downgrade) | MD (95%CI): -2.95 (-6.04, -0.09) | No head-to-head study and no heterogeneity.  There is no direct comparison between the interventions. | The included studies used different diagnostic criteria for septic shock. (downgrade) | Comprehensive search strategy. | Low |
| XBJ+WM VS WM | 100% of the estimate from studies at “some concerns” (downgrade) | MD (95%CI): -3.11 (-5.01, -1.47) | Mild heterogeneity according to *I*^2^=33.2%.  There is no indirect comparison between the interventions. | All the studies used the same diagnostic criterion for septic shock (Sepsis 3.0). | Comprehensive search strategy. | Moderate |
| SF+WM VS WM | 100% of the estimate from studies at “some concerns” (downgrade) | MD (95%CI): -0.16 (-2.57, 2.31) (downgrade) | Mild heterogeneity according to *I*^2^=0.0%.  There is no indirect comparison between the interventions. | The included studies used different diagnostic criteria for septic shock. (downgrade) | Comprehensive search strategy. | Very low |
| Ranking of treatment | 100% of the estimate from studies at “some concerns” (downgrade) | Ranking probability of SUCRA suggested precision in a ranking of treatments. | Mild heterogeneity in network meta-analyses according to global *I*^2^=14.1%.  There is no closed loop among the interventions. | The included studies used different diagnostic criteria for septic shock. (downgrade) | The funnel chart shows no obvious publication bias. | Low |

*Note: WM, Western Medicine; SF, Shenfu injection; SQFZ, Shenqifuzheng injection; SGM, Shengmai injection; XBJ, Xuebijing injection; SUCRA, surface under the cumulative ranking area curves.*

**9.4 Evaluation of the quality of evidence using GRADE framework for SOFA score at day 7 after interventions.**

| **Comparison** | **Study limitation** | **Imprecision** | **Heterogeneity and inconsistency** | **Indirectness** | **Publication bias** | **Confidence in MD for SOFA score at day 7 after interventions** |
| --- | --- | --- | --- | --- | --- | --- |
| XBJ+WM VS SM+WM | 100% of the estimate from studies at “some concerns” (downgrade) | MD (95%CI): -0.63 (-3.29, 2.01) (downgrade) | No head-to-head study and no heterogeneity.  There is no direct comparison between the interventions. | All the studies used the same diagnostic criterion for septic shock (Sepsis 2.0). | Comprehensive search strategy. | Low |
| XBJ+WM VS SQFZ+WM | 100% of the estimate from studies at “some concerns” (downgrade) | MD (95%CI): -0.94 (-3.73, 1.84) (downgrade) | No head-to-head study and no heterogeneity.  There is no direct comparison between the interventions. | The included studies used different diagnostic criteria for septic shock. (downgrade) | Comprehensive search strategy. | Very low |
| XBJ+WM VS SF+WM | 100% of the estimate from studies at “some concerns” (downgrade) | MD (95%CI): -1.47 (-2.88, -0.06) | No head-to-head study and no heterogeneity.  There is no direct comparison between the interventions. | All the studies used the same diagnostic criterion for septic shock (Sepsis 2.0). | Comprehensive search strategy. | Moderate |
| XBJ+WM VS WM | 100% of the estimate from studies at “some concerns” (downgrade) | MD (95%CI): -2.83 (-3.86, -1.82) | Severe heterogeneity according to *I*^2^=92.9%. (downgrade)  There is no indirect comparison between the interventions. | All the studies used the same diagnostic criterion for septic shock (Sepsis 2.0). | Comprehensive search strategy. | Low |
| SM+WM VS SQFZ+WM | 100% of the estimate from studies at “some concerns” (downgrade) | MD (95%CI): -0.31 (-3.87, 3.24) (downgrade) | No head-to-head study and no heterogeneity.  There is no direct comparison between the interventions. | The included studies used different diagnostic criteria for septic shock. (downgrade) | Comprehensive search strategy. | Very low |
| SM+WM VS SF+WM | 100% of the estimate from studies at “some concerns” (downgrade) | MD (95%CI): -0.84 (-3.46, 1.80) (downgrade) | No head-to-head study and no heterogeneity.  There is no direct comparison between the interventions. | All the studies used the same diagnostic criterion for septic shock (Sepsis 2.0). | Comprehensive search strategy. | Low |
| SM+WM VS WM | 100% of the estimate from studies at “some concerns” (downgrade) | MD (95%CI): -2.20 (-4.65, 0.25) (downgrade) | No head-to-head studies of 2 or more and no heterogeneity.  There is no indirect comparison between the interventions. | All the studies used the same diagnostic criterion for septic shock (Sepsis 2.0). | Comprehensive search strategy. | Low |
| SQFZ+WM VS SF+WM | 100% of the estimate from studies at “some concerns” (downgrade) | MD (95%CI): -0.53 (-3.28, 2.24) (downgrade) | No head-to-head study and no heterogeneity.  There is no direct comparison between the interventions. | The included studies used different diagnostic criteria for septic shock. (downgrade) | Comprehensive search strategy. | Very low |
| SQFZ+WM VS WM | 100% of the estimate from studies at “some concerns” (downgrade) | MD (95%CI): -1.89 (-4.48, 0.69) (downgrade) | No head-to-head studies of 2 or more and no heterogeneity.  There is no indirect comparison between the interventions. | All the studies used the same diagnostic criterion for septic shock (Sepsis 3.0). | Comprehensive search strategy. | Low |
| SF+WM VS WM | 100% of the estimate from studies at “some concerns” (downgrade) | MD (95%CI): -1.36 (-2.34, -0.40) | Mild heterogeneity according to *I*^2^=48.8%.  There is no indirect comparison between the interventions. | All the studies used the same diagnostic criterion for septic shock (Sepsis 2.0). | Comprehensive search strategy. | Moderate |
| Ranking of treatment | 100% of the estimate from studies at “some concerns” (downgrade) | Ranking probability of SUCRA suggested precision in a ranking of treatments. | Severe heterogeneity in network meta-analyses according to global *I*^2^=91.1%. (downgrade)  There is no closed loop among the interventions. | The included studies used different diagnostic criteria for septic shock. (downgrade) | The funnel chart shows existence of potential publication bias. (downgrade) | Very low |

*Note: WM, Western Medicine; SF, Shenfu injection; SM, Shenmai injection; SQFZ, Shenqifuzheng injection; XBJ, Xuebijing injection; SUCRA, surface under the cumulative ranking area curves.*

**9.5 Evaluation of the quality of evidence using GRADE framework for procalcitonin level at day 7 after interventions.**

| **Comparison** | **Study limitation** | **Imprecision** | **Heterogeneity and inconsistency** | **Indirectness** | **Publication bias** | **Confidence in MD for procalcitonin level at day 7 after interventions** |
| --- | --- | --- | --- | --- | --- | --- |
| XYP+WM VS SM+WM | 100% of the estimate from studies at “some concerns” (downgrade) | MD (95%CI): -34.29 (-38.75, -29.76) | No head-to-head study and no heterogeneity.  There is no direct comparison between the interventions. | The included studies used different diagnostic criteria for septic shock. (downgrade) | Comprehensive search strategy. | Low |
| XYP+WM VS SGM+WM | 100% of the estimate from studies at “some concerns” (downgrade) | MD (95%CI): -35.46 (-40.01, -30.89) | No head-to-head study and no heterogeneity.  There is no direct comparison between the interventions. | All the studies used the same diagnostic criterion for septic shock (Sepsis 2.0). | Comprehensive search strategy. | Moderate |
| XYP+WM VS DS+WM | 100% of the estimate from studies at “some concerns” (downgrade) | MD (95%CI): -35.56 (-40.51, -30.59) | No head-to-head study and no heterogeneity.  There is no direct comparison between the interventions. | All the studies used the same diagnostic criterion for septic shock (Sepsis 2.0). | Comprehensive search strategy. | Moderate |
| XYP+WM VS SF+WM | 100% of the estimate from studies at “some concerns” (downgrade) | MD (95%CI): -35.87 (-40.13, -31.62) | No head-to-head study and no heterogeneity.  There is no direct comparison between the interventions. | All the studies used the same diagnostic criterion for septic shock (Sepsis 2.0). | Comprehensive search strategy. | Moderate |
| XYP+WM VS XBJ+WM | 100% of the estimate from studies at “some concerns” (downgrade) | MD (95%CI): -36.27 (-40.66, -31.93) | No head-to-head study and no heterogeneity.  There is no direct comparison between the interventions. | The included studies used different diagnostic criteria for septic shock. (downgrade) | Comprehensive search strategy. | Low |
| XYP+WM VS WM | 100% of the estimate from studies at “some concerns” (downgrade) | MD (95%CI): -37.26 (-41.38, -33.13) | No head-to-head studies of 2 or more and no heterogeneity.  There is no indirect comparison between the interventions. | All the studies used the same diagnostic criterion for septic shock (Sepsis 2.0). | Comprehensive search strategy. | Moderate |
| SM+WM VS SGM+WM | 100% of the estimate from studies at “some concerns” (downgrade) | MD (95%CI): -1.17 (-3.86, 1.44) (downgrade) | No head-to-head study and no heterogeneity.  There is no direct comparison between the interventions. | The included studies used different diagnostic criteria for septic shock. (downgrade) | Comprehensive search strategy. | Very low |
| SM+WM VS DS+WM | 100% of the estimate from studies at “some concerns” (downgrade) | MD (95%CI): -1.27 (-4.61, 1.99) (downgrade) | No head-to-head study and no heterogeneity.  There is no direct comparison between the interventions. | The included studies used different diagnostic criteria for septic shock. (downgrade) | Comprehensive search strategy. | Very low |
| SM+WM VS SF+WM | 100% of the estimate from studies at “some concerns” (downgrade) | MD (95%CI): -1.59 (-3.71, 0.46) (downgrade) | No head-to-head study and no heterogeneity.  There is no direct comparison between the interventions. | The included studies used different diagnostic criteria for septic shock. (downgrade) | Comprehensive search strategy. | Very low |
| SM+WM VS XBJ+WM | 100% of the estimate from studies at “some concerns” (downgrade) | MD (95%CI): -1.97 (-4.36, 0.23) (downgrade) | No head-to-head study and no heterogeneity.  There is no direct comparison between the interventions. | The included studies used different diagnostic criteria for septic shock. (downgrade) | Comprehensive search strategy. | Very low |
| SM+WM VS WM | 100% of the estimate from studies at “some concerns” (downgrade) | MD (95%CI): -2.97 (-4.81, -1.21) | Severe heterogeneity according to *I*^2^=94.2%. (downgrade)  There is no indirect comparison between the interventions. | The included studies used different diagnostic criteria for septic shock. (downgrade) | Comprehensive search strategy. | Very low |
| SGM+WM VS DS+WM | 100% of the estimate from studies at “some concerns” (downgrade) | MD (95%CI): -0.10 (-3.48, 3.28) (downgrade) | No head-to-head study and no heterogeneity.  There is no direct comparison between the interventions. | All the studies used the same diagnostic criterion for septic shock (Sepsis 2.0). | Comprehensive search strategy. | Low |
| SGM+WM VS SF+WM | 100% of the estimate from studies at “some concerns” (downgrade) | MD (95%CI): -0.42 (-2.63, 1.81) (downgrade) | No head-to-head study and no heterogeneity.  There is no direct comparison between the interventions. | All the studies used the same diagnostic criterion for septic shock (Sepsis 2.0). | Comprehensive search strategy. | Low |
| SGM+WM VS XBJ+WM | 100% of the estimate from studies at “some concerns” (downgrade) | MD (95%CI): -0.80 (-3.28, 1.55) (downgrade) | No head-to-head study and no heterogeneity.  There is no direct comparison between the interventions. | The included studies used different diagnostic criteria for septic shock. (downgrade) | Comprehensive search strategy. | Very low |
| SGM+WM VS WM | 100% of the estimate from studies at “some concerns” (downgrade) | MD (95%CI): -1.80 (-3.76, 0.15) (downgrade) | Moderate  heterogeneity according to *I*^2^=74.3%. (downgrade)  There is no indirect comparison between the interventions. | All the studies used the same diagnostic criterion for septic shock (Sepsis 2.0). | Comprehensive search strategy. | Very low |
| DS+WM VS SF+WM | 100% of the estimate from studies at “some concerns” (downgrade) | MD (95%CI): -0.32 (-3.27, 2.64) (downgrade) | No head-to-head study and no heterogeneity.  There is no direct comparison between the interventions. | All the studies used the same diagnostic criterion for septic shock (Sepsis 2.0). | Comprehensive search strategy. | Low |
| DS+WM VS XBJ+WM | 100% of the estimate from studies at “some concerns” (downgrade) | MD (95%CI): -0.70 (-3.87, 2.35) (downgrade) | No head-to-head study and no heterogeneity.  There is no direct comparison between the interventions. | The included studies used different diagnostic criteria for septic shock. (downgrade) | Comprehensive search strategy. | Very low |
| DS+WM VS WM | 100% of the estimate from studies at “some concerns” (downgrade) | MD (95%CI): -1.70 (-4.47, 1.06) (downgrade) | No head-to-head studies of 2 or more and no heterogeneity.  There is no indirect comparison between the interventions. | No report on which diagnostic criteria for septic shock were used. (downgrade) | Comprehensive search strategy. | Very low |
| SF+WM VS XBJ+WM | 100% of the estimate from studies at “some concerns” (downgrade) | MD (95%CI): -0.39 (-2.22, 1.32) (downgrade) | No head-to-head study and no heterogeneity.  There is no direct comparison between the interventions. | The included studies used different diagnostic criteria for septic shock. (downgrade) | Comprehensive search strategy. | Very low |
| SF+WM VS WM | 100% of the estimate from studies at “some concerns” (downgrade) | MD (95%CI): -1.38 (-2.44, -0.34) | Severe heterogeneity according to *I*^2^=95.8%. (downgrade)  There is no indirect comparison between the interventions. | All the studies used the same diagnostic criterion for septic shock (Sepsis 2.0). | Comprehensive search strategy. | Low |
| XBJ+WM VS WM | 100% of the estimate from studies at “some concerns” (downgrade) | MD (95%CI): -1.00 (-2.37, 0.48) (downgrade) | Severe heterogeneity according to *I*^2^=86.9%. (downgrade)  There is no indirect comparison between the interventions. | The included studies used different diagnostic criteria for septic shock. (downgrade) | Comprehensive search strategy. | Very low |
| Ranking of treatment | 100% of the estimate from studies at “some concerns” (downgrade) | Ranking probability of SUCRA suggested precision in a ranking of treatments. | Severe heterogeneity in network meta-analyses according to global *I*^2^=99.6%. (downgrade)  There is no closed loop among the interventions. | The included studies used different diagnostic criteria for septic shock. (downgrade) | The funnel chart shows no obvious publication bias. | Very low |

*Note: WM, Western Medicine; XYP, Xiyanping injection; SF, Shenfu injection; SM, Shenmai injection; SGM, Shengmai injection; XBJ, Xuebijing injection; DS, Danshen injection; SUCRA, surface under the cumulative ranking area curves.*

**9.6 Evaluation of the quality of evidence using GRADE framework for serum lactate level at day 7 after interventions.**

| **Comparison** | **Study limitation** | **Imprecision** | **Heterogeneity and inconsistency** | **Indirectness** | **Publication bias** | **Confidence in MD for serum lactate level at day 7 after interventions** |
| --- | --- | --- | --- | --- | --- | --- |
| SM+WM VS SF+WM | 100% of the estimate from studies at “some concerns” (downgrade) | MD (95%CI): -0.12 (-0.89, 0.64) (downgrade) | No head-to-head study and no heterogeneity.  There is no direct comparison between the interventions. | All the studies used the same diagnostic criterion for septic shock (Sepsis 2.0). | Comprehensive search strategy. | Low |
| SM+WM VS XBJ+WM | 100% of the estimate from studies at “some concerns” (downgrade) | MD (95%CI): -0.99 (-1.81, -0.16) | No head-to-head study and no heterogeneity.  There is no direct comparison between the interventions. | All the studies used the same diagnostic criterion for septic shock (Sepsis 2.0). | Comprehensive search strategy. | Moderate |
| SM+WM VS WM | 100% of the estimate from studies at “some concerns” (downgrade) | MD (95%CI): -1.43 (-2.08, -0.79) | Severe heterogeneity according to *I*^2^=83.9%. (downgrade)  There is no indirect comparison between the interventions. | All the studies used the same diagnostic criterion for septic shock (Sepsis 2.0). | Comprehensive search strategy. | Low |
| SF+WM VS XBJ+WM | 100% of the estimate from studies at “some concerns” (downgrade) | MD (95%CI): -0.86 (-1.52, -0.20) | No head-to-head study and no heterogeneity.  There is no direct comparison between the interventions. | All the studies used the same diagnostic criterion for septic shock (Sepsis 2.0). | Comprehensive search strategy. | Moderate |
| SF+WM VS WM | 100% of the estimate from studies at “some concerns” (downgrade) | MD (95%CI): -1.31 (-1.72, -0.89) | Severe heterogeneity according to *I*^2^=93.6% (downgrade)  There is no indirect comparison between the interventions. | All the studies used the same diagnostic criterion for septic shock (Sepsis 2.0). | Comprehensive search strategy. | Low |
| XBJ+WM VS WM | 100% of the estimate from studies at “some concerns” (downgrade) | MD (95%CI): -0.44 (-0.96, 0.07) (downgrade) | Severe heterogeneity according to *I*^2^=84.8% (downgrade)  There is no indirect comparison between the interventions. | All the studies used the same diagnostic criterion for septic shock (Sepsis 2.0). | Comprehensive search strategy. | Very low |
| Ranking of treatment | 100% of the estimate from studies at “some concerns” (downgrade) | Ranking probability of SUCRA suggested precision in a ranking of treatments. | Severe heterogeneity in network meta-analyses according to global *I*^2^=93.5%. (downgrade)  There is no closed loop among the interventions. | All the studies used the same diagnostic criterion for septic shock (Sepsis 2.0). | The funnel chart shows existence of potential publication bias. (downgrade) | Very low |

*Note: WM, Western Medicine; SF, Shenfu injection; SM, Shenmai injection; XBJ, Xuebijing injection; SUCRA, surface under the cumulative ranking area curves.*
